# Electric‐Field‐Driven Spin Resonance by On‐Surface Exchange Coupling to a Single‐Atom Magnet

**DOI:** 10.1002/advs.202302033

**Published:** 2023-07-19

**Authors:** Soo‐hyon Phark, Hong Thi Bui, Alejandro Ferrón, Joaquin Fernández‐Rossier, Jose Reina‐Gálvez, Christoph Wolf, Yu Wang, Kai Yang, Andreas J. Heinrich, Christopher P. Lutz

**Affiliations:** ^1^ Center for Quantum Nanoscience Institute for Basic Science (IBS) Seoul 03760 Republic of Korea; ^2^ Department of Physics Ewha Womans University Seoul 03760 Republic of Korea; ^3^ IBM Research Division Almaden Research Center San Jose CA 95120 USA; ^4^ Instituto de Modelado e Innovación Tecnológica (CONICET‐UNNE) and Facultad de Ciencias Exactas Naturales y Agrimensura Universidad Nacional del Nordeste Avenida Libertad 5400 Corrientes W3404AAS Argentina; ^5^ International Iberian Nanotechnology Laboratory (INL) Braga 4715‐330 Portugal; ^6^ Beijing National Laboratory for Condensed Matter Physics and Institute of Physics Chinese Academy of Sciences Beijing 100864 China

**Keywords:** atom manipulation, electron spin resonance, Rabi rate, scanning tunneling microscopy, single spin qubit, single‐atom magnet

## Abstract

Coherent control of individual atomic and molecular spins on surfaces has recently been demonstrated by using electron spin resonance (ESR) in a scanning tunneling microscope (STM). Here, a combined experimental and modeling study of the ESR of a single hydrogenated Ti atom that is exchange‐coupled to a Fe adatom positioned 0.6–0.8 nm away by means of atom manipulation is presented. Continuous wave and pulsed ESR of the Ti spin show a Rabi rate with two contributions, one from the tip and the other from the Fe, whose spin interactions with Ti are modulated by the radio‐frequency electric field. The Fe contribution is comparable to the tip, as revealed by its dominance when the tip is retracted, and tunable using a vector magnetic field. The new ESR scheme allows on‐surface individual spins to be addressed and coherently controlled without the need for magnetic interaction with a tip. This study establishes a feasible implementation of spin‐based multi‐qubit systems on surfaces.

## Graphical abstract

A sophisticated method is established to drive electron spin resonance of a single spin on the surface using single‐atom magnets in proximity and the working principles are disclosed. This provides a key protocol to coherently control spins outside the tunnel junction of a scanning tunneling microscope, allowing on‐surface multi‐spin qubit platforms.

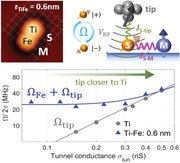

## 1 Introduction

Atomic and molecular spins on surfaces can provide a solid‐state qubit platform^[^ ^1^, ^2^ ^]^ that is unique in allowing bottom‐up design capabilities. The coherent manipulations of single spins in a scanning tunneling microscope (STM) equipped with pulsed electron spin resonance (ESR) opened a way to utilize individual atomic^[^ ^3^, ^4^ ^]^ and molecular^[^ ^5^ ^]^ spins on surfaces as feasible qubits for quantum information and computation. To date, magnetic resonance in STM^[^ ^6^, ^7^, ^8^, ^9^, ^10^ ^]^ has relied on the electric‐field‐driven modulation of the atomic scale magnetic interaction between the spin on the surface and the magnetic STM tip.^[^ ^11^, ^12^, ^13^ ^]^ A coherence time of a few tens of ns has been reported for the spins in the tunnel junction.^[^ ^3^, ^5^, ^9^ ^]^ This coherence is mainly limited by the scattering of the spin states by tunneling electrons^[^ ^5^, ^14^ ^]^ and by the fluctuating magnetic field due to tip vibrations, limiting the number of available quantum gate operations within the coherence time. Moreover, such a “tip‐field‐driven ESR” allows only one spin in the tunnel junction to be coherently controlled at a time, which has so far prohibited quantum manipulation of multiple spin qubits in STM. Therefore, it is necessary to develop a new mechanism to drive the ESR of a spin in STM, free from strong coupling to the tip's magnetic field. Such a scheme has recently been realized with the demonstration of coherent control of spins that are outside the tunnel junction, which showed a much‐enhanced coherence time.^[^ ^15^ ^]^ Here, we show an elaborated study of the mechanism that enables the coherent control of such remote spins.

In this work, we present continuous wave (CW) and pulsed ESR of hydrogenated Ti atoms (spin *S* = 1/2) adsorbed on a thin MgO film,^[^ ^16^ ^]^ with Fe atoms located in close proximity, in an ultra‐high vacuum STM operating at 1.2 K. Pulsed ESR of Ti atoms in such atomic pairs showed large Rabi rates, up to Ω/2*π* ≈ 25 MHz, even when the tip‐Ti distance was large, which are comparable with the rates when driven by the interaction with the tip magnetic moment.^[^ ^3^, ^4^, ^5^ ^]^ CW‐ESR on the Ti‐Fe pair with an interatomic separation of 0.6–0.8 nm in a vector magnetic field revealed that the spin‐spin interaction ranges up to 20 GHz and the driving strength is tunable as well. Combined with a model study, we show that such a driving field stems from the Fe atom's magnetic field gradient. The inhomogeneous local magnetic field provided by such a single‐atom magnet is thus able to replace the role of the magnetic tip in the coherent driving of a qubit in an ESR‐STM.^[^ ^7^, ^10^, ^11^, ^12^, ^13^, ^17^ ^]^

## 2 Results

**Figure 1:**
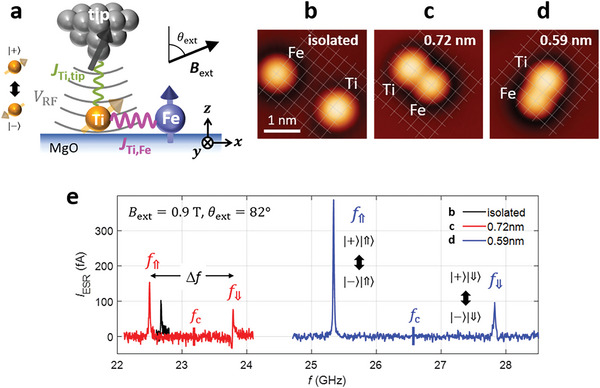
Electron spin resonance in Ti‐Fe pairs. a) Schematic showing a Ti spin (*S* = 1/2) coupled with Fe on MgO in electron spin resonance. *J*_Ti,Fe_ and *J*_Ti,tip_ are Ti‐Fe and Ti‐tip spin‐spin interactions, which drive the ESR of the Ti spin when coupled with the RF voltage *V*_RF_ applied to the tunnel junction. b–d) STM images of Ti atoms with Fe positioned at distances of 1.9, 0.72, and 0.59 nm away from Ti, respectively (*V*_DC_ = 50 mV, setpoint tunnel current *I*_tun_ = 10 pA). e) Continuous wave ESR spectra measured on Ti atoms in b) (black), c) (red), and d) (blue) at *B*_ext_ = 0.9 T with the polar angle *θ*_ext_ = 82^o^ (*V*_DC_ = 50 mV, *V*_RF_ = 20 mV, *I*_tun_ = 15 pA, *T* = 1.2 K). The frequency *f*_c_ indicates the center of the two peaks in each curve.

Our ESR‐STM experiment on a pair of Ti and Fe atoms, hereafter referred to as a “Ti‐Fe pair”, is illustrated in **Figure** **1**a. Both the tip and the Fe are coupled with the Ti spin via exchange interactions, *J*_Ti,tip_ and *J*_Ti,Fe_, respectively. The radio‐frequency (RF) voltage *V*_RF_ applied between the tip and sample drives a spin resonance of the Ti, whose influence on the spin population is measured by the spin‐polarized tunneling current (see Experimental Section).^[^ ^6^ ^]^ By utilizing atom manipulation techniques,^[^ ^18^ ^]^ we constructed such Ti‐Fe pairs of three different separations (Figure 1b–d) and performed ESR on the Ti atom of each pair as shown in Figure 1e.

The ESR spectrum from the pair with the largest separation shown in Figure 1b (black curve) exhibited only one resonance peak, determined by the Zeeman energy of an isolated Ti,^[^ ^7^, ^10^ ^]^ which indicates a vanishingly small interaction with Fe. In contrast, Ti‐Fe pairs of smaller separations (Figure 1c,d) showed a clear splitting (Δ*f*) of the resonance peak (red and blue curves in Figure 1e), indicating a sizable magnetic interaction between Ti and Fe in the pair. This splitting was largest for the pair with the smaller separation (Figure S1, Supporting Information).

The Fe spin fluctuates between spin up (|⇑〉) and down (|⇓〉) states, corresponding to its expectation value along the ***z*** direction (surface normal, see Figure 1a) 〈*S*_z_〉 = +2 and −2, respectively, due to its large out‐of‐plane uniaxial magnetic anisotropy energy on the MgO surface (Figure S4 and Text S2.3, Supporting Information).^[^ ^20^ ^]^ The switching between those two Fe spin states is slow enough that the resonance of the Ti spin (*S* = 1/2) at each given Fe state is well‐defined for the Ti spin's two eigenstates (|⇑〉) and down (|⇓〉) along its quantization axis as determined by the total static field ***B***_0_. Following the experimental configuration sketched in Figure 1a, ***B***_0_ is determined by the vector sum of three contributions: the external field (***B***_ext_), tip‐induced field (***B***_tip_), and Fe‐induced field (***B***_Fe_) (also see Figure 3c). Hence, the resonances of the Ti spin are resolved in the ESR spectrum as two individual peaks, well separated by the Fe‐Ti interaction energy.^[^ ^19^ ^]^ The quasi‐static behavior of Fe is consistent with isolated Fe atoms on MgO and caused by its spin relaxation time of a few tens of microseconds at the magnetic fields used here,^[^ ^21^, ^22^ ^]^ which is longer by about 2–3 orders of magnitude than the typical time scale for coherence and coherent manipulation of the Ti spin.^[^ ^15^ ^]^ As the Ti and Fe were positioned closer together, we observed significant shifts in the center frequency *f*_c_ of resonance peak pairs by up to a few GHz. This shift arises from the change of the Ti spin direction due to the Ti‐Fe interaction, which increases the Ti Zeeman energy due to the anisotropy of its *g*‐factor (Figure S6, Supporting Information).^[^ ^10^ ^]^

**Figure 2:**
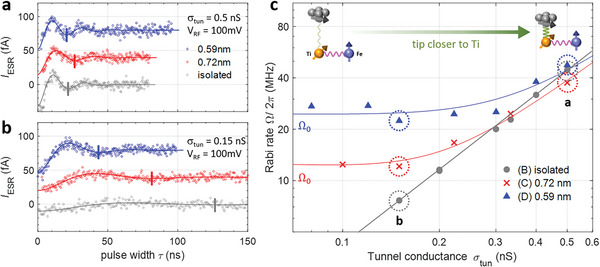
Influence of Fe on the Rabi rate. a,b) Rabi oscillations using pulsed ESR on Ti atoms shown in Figure 1b–d at junction conductance of a) 0.5 and b) 0.15 nS, *V*_RF_ = 100 mV. The oscillation period of each curve is marked by the vertical bar. The curves are successively shifted vertically by 40 fA for clarity. c) Rabi rate Ω extracted from Rabi oscillation measurements of isolated Ti (gray circles), Ti‐Fe of 0.72 nm (red crosses), and Ti‐Fe of 0.59 nm (blue triangles) (*I*_tun_ = 10 pA, *V*_RF_ = 100 mV, *T* = 1.2 K, *B*_ext_ = 0.9 T, *θ*_ext_ = 82^o^). The dotted circles denote the data points corresponding to the curves in (a,b) with the same color codes. Two insets illustrate the magnetic interactions, *J*_Ti,Fe_ and *J*_Ti,tip_, for small (left) and large (right) tunnel conductance regimes, respectively. Solid curves are fits using the model described in Text S1, Supporting Information, resulting in zero conductance Rabi rates Ω_0_/2*π* of 27 ± 2 and 14 ± 2MHz for the pairs of 0.59 and 0.72 nm, respectively.

Similar to the role of the exchange field from the Ti‐tip interaction *J*_Ti,tip_ in ESR of an isolated single spin,^[^ ^11^, ^12^, ^13^ ^]^ the Ti‐Fe interaction *J*_Ti,Fe_ can also effectively generate an inhomogeneous magnetic field at the location of Ti, which is able to drive the spin resonance of Ti. To demonstrate that the Ti‐Fe interaction can indeed drive the ESR of Ti, we performed pulsed ESR on the Ti atoms in each of the three Ti‐Fe pairs shown in Figure 1b–d and measured their Rabi rates (Ω) as a function of tunnel conductance (*σ*_tun_) by varying the tip‐Ti distance (**Figure** **2** and Figure S7, Supporting Information). At a large tunnel conductance, the measured Rabi rates were nearly independent of the Ti‐Fe distance showing that driving was dominated by the Ti‐tip interaction (Figure 2a). In contrast, at small tunnel conductance, we found a strong dependence of the Rabi rate on the Ti‐Fe distance (Figure 2b). The Rabi rate for the largest Ti‐Fe distance (effectively an isolated Ti atom) showed a linear decrease for a decreasing tunnel conductance (gray in Figure 2c) that extrapolates to Ω ≈ 0 at zero tunnel conductance, which implies a vanishing driving field at a very large tip‐atom distance. This linear behavior indicates an exponential dependence of the driving field on the tip height, a fingerprint for the exponential nature of the driving field contributed by the Ti‐tip exchange interaction as previously observed.^[^ ^12^ ^]^

In contrast, the Rabi rate of Ti positioned at sub‐nanometer distances to Fe (Figure 1c,d) remained finite, approaching a limiting value Ω_0_ (Figure 2c). This strongly suggests that the ESR of Ti was driven effectively by the presence of the Fe spin at a tip height where the driving magnetic field from the tip was negligible. The driving RF electric field due to the presence of the tip remains present at all tip heights tested and decays only slowly (with inverse distance) as the tip is withdrawn from the Ti. Furthermore, we found that the zero conductance Rabi rate measured on the pair with a separation of 0.72 nm was a factor of ≈2 smaller than that of the pair with 0.59 nm. The Rabi rate saturated at Ω_0_/2*π* = 25.0 ± 6.4MHz) at *V*_RF_ = 100 mV for the closer pair (Figures S8–S10, Supporting Information), which is sufficient to coherently invert the spin state in  20ns.^[^ ^3^, ^4^, ^5^ ^]^ We note that this Rabi rate is comparable to that of the isolated Ti for the closest tip‐Ti distances tested.^[^ ^3^, ^4^ ^]^ This characteristic dependence of the Rabi rates on the tunnel conductance demonstrates that a single‐atom magnet is indeed able to coherently drive the ESR of a single‐atom spin nearby.

**Figure 3:**
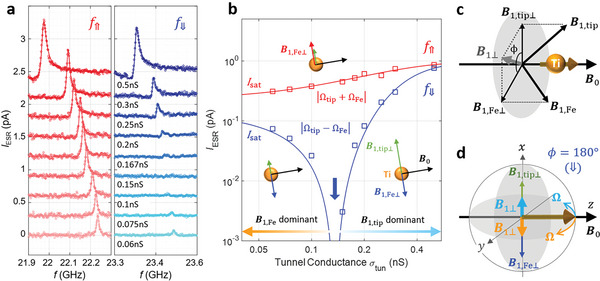
Crossover from tip‐driven to Fe‐driven ESR. a) CW‐ESR spectra measured on the Ti‐Fe of 0.72 nm at frequency ranges across the peaks $f_{⇑}$ (red) and $f_{⇓}$ (blue) for a varying tunnel conductance (*I*_tun_ = 15 pA, *V*_RF_ = 20 mV, *T* = 1.2 K, *B*_ext_ = 0.9 T, *θ*_ext_ = 82^o^). The solid curves are asymmetric Lorentzian fits. The curves are successively shifted vertically by 0.3 pA for clarity. b) Peak height *I*_ESR_ versus tunnel conductance extracted from CW‐ESR spectra in (a). Fits according to the discussion in the main text are overlaid (solid curves; see Section 3). Extrapolation of the fit curves to zero conductance gives intercepts *I*_sat_ = 0.226 pA ($f_{⇑}$) and 0.142 pA ($f_{⇓}$). The three insets illustrate the vectorial relationships between the ESR driving fields contributed from the Fe (red or blue) and the tip (light green). c) Vectorial relationship of driving fields from Fe (***B***_1,Fe_) and tip (***B***_1,tip_). ***B***_0_ is the total static field at the Ti position, composed of external (***B***_ext_), tip‐induced field (***B***_tip_), and Fe‐induced (***B***_Fe_) fields. ***B***_1,Fe⊥_ and ***B***_1,tip⊥_ denote projections of ***B***_1,Fe_ and ***B***_1,tip_, respectively, to a plane perpendicular to the total static field ***B***_0_. d) A schematic of the Bloch sphere in a condition that ***B***_1,Fe⊥_ and ***B***_1,tip⊥_ are antiparallel (*ϕ* = 180^o^) and showing resultant Rabi rotations of the Bloch vector of the Ti spin (brown thick arrow). The net driving field ***B***_1⊥_ changes its direction depending on the magnitude of the tip‐induced driving field ***B***_1,tip⊥_, leading to corresponding change in the Rabi rotation.

For a given Ti‐Fe pair, we can further discriminate between the influence of the tip spin and that of the Fe spin on the ESR of Ti by examining the dependence of the Rabi rate on the tip height. Contribution from the tip to the Rabi rate is tunable by the tip‐Ti distance, while that from the Fe is essentially independent of the tip‐Ti distance. **Figure** **3**a shows an evolution of CW ESR spectra as a function of tunnel conductance obtained from the pair with a separation of 0.72 nm. Here we make use of the CW‐ESR to explore cases where the Rabi rate is too small to measure directly in the time domain. The ESR peak height *I*_ESR_ is closely related to the Rabi rate according to *I*_ESR_ ∝ Ω^2^ *T*_1_ *T*_2_/(1 + Ω^2^ *T*_1_ *T*_2_), where *T*_1_ is the energy relaxation time and *T*_2_ is the dephasing time (Supporting Information). The $f_{⇑}$ peak (the peak due to Fe in the spin‐up state) showed a monotonic decrease of its height with decreasing conductance, reflecting a decreasing contribution of the tip spin to the driving field as the tip was moved away from the Ti atom; however, the peak height approached a non‐zero limiting value as the tunnel conductance approached zero. In contrast, the height of the $f_{⇓}$ peak vanished at an intermediate tunnel conductance of about 0.15 nS and reappeared as the tip was withdrawn further from the atom. This behavior differs markedly from the ESR peak heights of isolated Ti atoms (Figure S12, Supporting Information) or Ti‐Ti pairs,^[^ ^9^, ^16^, ^23^ ^]^ where the heights decrease monotonically and vanish at a very low conductance.

The vanishing ESR peak at $f_{⇓}$ results from a cancellation of the two Rabi rates contributed from the tip and Fe spins at a critical tip height, as follows: ESR of Ti in a Ti‐Fe pair is driven by a vector sum of two driving fields, one stemming from the interactions with the tip and the other with the Fe single‐atom magnet. This is illustrated in the three insets in Figure 3b. When the two driving fields are antiparallel, they can cancel each other and result in the disappearance of the peak for $f_{⇓}$ at a critical tip height, here corresponding to a conductance of 0.15 nS. On the other hand, the opposite orientation of the Fe spin leads to a parallel alignment of the two, which explains the monotonic decrease but the non‐vanishing peak height for $f_{⇑}$ as the tunnel conductance approaches zero. This crossover demonstrates the presence of two competing transverse RF driving fields. Quantitative modeling of these summed driving fields gives an excellent agreement with the measurement (Figure 3b–d).

**Figure 4:**
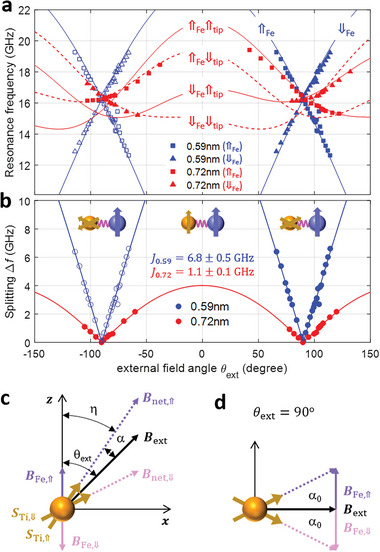
Angle dependence of ESR frequencies on the applied magnetic field. a) ESR resonance peak frequencies and b) splitting Δ*f* in CW‐ESR spectra measured from Ti‐Fe pairs of 0.72 nm (red) and 0.59 nm (blue) at a varying polar angle (*θ*_ext_) of ***B***_ext_. Solid curves are fits using the model described in Equations (1) and (2). Red solid (dashed) curves correspond to the up (down) state of the tip spin. (*V*_DC_ = 200 mV, *I*_tun_ = 10 pA, *V*_RF_ = 30 mV for the 0.59 nm pair; *V*_DC_ = 30 mV, *I*_tun_ = 20 pA, *V*_RF_ = 30 mV for the 0.72 nm pair; *T* = 0.4 K, *B*_ext_ = 0.6 T). c,d) Schemes of the net magnetic field ***B***_net_ at the Ti position, composed of the external field ***B***_ext_ and Fe‐induced field ***B***_Fe_ in the plane of ***B***_ext_ vector with c) an arbitrary and d) 90^o^ field angle *θ*_ext_. *α* and *α*_0_ denote the angles added by the Fe‐induced field. Here, the field induced by the tip magnetic field ***B***_tip_ is omitted to focus on the discussion only on the effect of ***B***_Fe_.

Our measurements have shown that the spin‐spin interaction in a Ti‐Fe pair is the origin of the finite Rabi rates when the Ti‐tip interaction is negligible as well as the peak splitting in the ESR spectra. To characterize the Ti‐Fe interaction in more detail, we measured the dependence of the peak splitting in the ESR spectra of the two pairs on the field angle (**Figure** **4**a,b and Figure S14, Supporting Information). An isotropic exchange type Ti‐Fe interaction *J*_Ti,Fe_ ***S***_Ti_· ***S***_Fe_, which depends only on the Fe and Ti spin directions, is controllable by the direction of the external magnetic field. The Fe spin orientation is quasi‐static and always aligned perpendicular to the MgO surface.^[^ ^6^, ^20^ ^]^ Figure 4a,b show the resonance peak frequencies and peak splitting, respectively, measured as a function of the angle between the magnetic field and the surface normal (*θ*_ext_).

The splitting in both pairs reaches a maximum when the field is normal to the surface (*θ*_ext_ = 0) and vanishes for in‐plane fields (*θ*_ext_ = +90^o^ or −90^o^), confirming that the Fe spin points along the surface are normal. The Fe spin generates an effective field ***B***_Fe_ at the position of the Ti atom, as depicted by ***B***_Fe⇑_ and ***B***_Fe⇓_ in Figure 4c, resulting in the net field ***B***_net_ = ***B***_ext_ + ***B***_Fe_, along which the Ti spin aligns (Supporting Information). The peak splitting (Δ*f* ) from the Ti‐Fe interaction is expected to show a cosine dependence on the angle between the two spins *η* = *θ*_ext_ − *α*, with *α* formed by ***B***_Fe_. The orientation of the Fe spin normal to the surface leads to a peak splitting of |*cosη*|, which is mirror‐symmetric about an in‐plane direction of ***B***_ext_. This accurately describes the features in the field angle dependence of the splitting in Figure 4b and suggests that the peak splitting indeed originates only from the Ti‐Fe interaction. Fitting the data in Figure 4b resulted in exchange couplings *J*_Ti,Fe_ = 1.1 ± 0.1 and 6.8 ± 0.5 GHz for the pairs of 0.72 and 0.59 nm, respectively.

**Figure 5:**
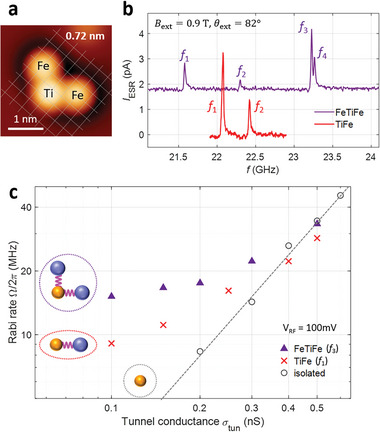
ESR of Ti using two Fe atoms. a) A Fe‐Ti‐Fe spin complex with Ti‐Fe separations of 0.72 nm. b) Continuous wave ESR spectra measured on Ti of the complex in (a) (purple) and a Ti‐Fe pair of 0.72 nm (red) (*V*_DC_ = 50 mV, *I*_tun_ = 15 pA, *V*_RF_ = 100 mV, *T* = 1.2 K, *B*_ext_ = 0.9 T, *θ*_ext_ = 82^o^). c) Rabi rates obtained from pulsed ESR of the complex (purple triangles) and Ti‐Fe pair of 0.72 nm (red crosses), and an isolated Ti (gray open circles) at a varying tunnel conductance. The insets schematically depict the Ti‐Fe interactions for three groups of Rabi rates (*V*_RF_ = 100 mV, *T* = 1.2 K, *B*_ext_ = 0.9 T, *θ*_ext_ = 82^o^).

Our experimental findings clearly show that a single‐atom magnet can indeed provide an atomic‐scale and reliable local transverse field, which can be used to coherently drive ESR of a nearby Ti spin with an oscillating electric field. To enhance the driving strength, a straightforward strategy is positioning more single‐atom magnets in proximity. To demonstrate this, we constructed a Fe‐Ti‐Fe structure by adding one more Fe atom near a Ti‐Fe pair of 0.72 nm, as shown in **Figure** **5**a, and performed ESR measurements on the Ti atom. The ESR spectrum from this spin complex showed four resonance peaks (Figure 5b), corresponding to four spin orientations of two Fe spins, |⇑⇑〉, |⇓⇑〉, |⇑⇓〉, and |⇓⇓〉 (Figure S5, Supporting Information). To figure out the effect of two Fe atoms on the ESR of the Ti spin, we performed pulsed ESR on this complex at the resonance peak *f*_3_ (Figure S17, Supporting Information). Figure 5C shows the Rabi rate as a function of the tunnel conductance (purple) as well as that measured on a Ti‐Fe pair with the same tip (red). This comparison directly reveals an enhancement of the Fe‐induced driving field by a factor of about two, indicating that our atomic‐scale and bottom‐up approach is plausible to tailor magnetic resonances in artificial atomic structures.

## 3 Discussion

To have a rigorous understanding of how the spin interactions influence the ESR spectra, we set up a time‐independent Hamiltonian for the spin configurations in this work as illustrated in Figure 1a.

(1) $${\hat{H}}_{0}=-\mu_{B}g_{\mathrm{Ti}}S_{\mathrm{Ti}}\cdot B_{\mathrm{ext}}+J_{\mathrm{Ti},\mathrm{Fe}}S_{\mathrm{Ti}}\cdot S_{\mathrm{Fe}}+J_{\mathrm{Ti},\mathrm{tip}}S_{\mathrm{Ti}}\cdot S_{\mathrm{tip}}$$

where *g*_Ti_ is the *g*‐factor of the Ti spin. According to the simplified models of the spin states of Fe (Figure S4, Supporting Information) and tip (Figure S15, Supporting Information),^[^ ^10^ ^]^ we treat the Fe and the tip's spins as classical magnetic moments of spin expectation values 〈 ***S***_Fe_〉 and 〈 ***S***_tip_〉 pointing along the directions ± ***z*** and ± ***n***_tip_, respectively. Here ***n**_tip_* = (*n_x_*,*n_y_*,*n_z_*) is determined by the uniaxial magnetic anisotropy of the tip magnetic moment. We obtained ***n***_tip_ from field‐angle (*θ*_ext_) dependence of ESR frequency, peak height, and Rabi rate measured on an isolated Ti atom (Figure S15, Supporting Information). Equation (1) can be reduced to a relatively simple form.

(2) $$\begin{array}{c} {\hat{H}}_{0}=S_{\mathrm{Ti}}\cdot {\tilde{B}}_{0},\\ {\tilde{B}}_{0}=\left(-g_{x}\mu_{B}B_{x}+J_{\mathrm{Ti},\mathrm{tip}}\langle S_{\mathrm{tip}}\rangle n_{x},-g_{y}\mu_{B}B_{y}+J_{\mathrm{Ti},\mathrm{tip}}\langle S_{\mathrm{tip}}\rangle n_{y}\right.\\ \;\;\;\left.-g_{z}\mu_{B}B_{z}+J_{\mathrm{Ti},\mathrm{tip}}\langle S_{\mathrm{tip}}\rangle n_{z}+J_{\mathrm{Ti},\mathrm{Fe}}\langle S_{\mathrm{Fe}}\rangle \right)\; \end{array}$$

where ${\tilde{B}}_{0}$ is a generalized magnetic field with four different cases, one for each sign of 〈 ***S***_Fe_〉 and 〈 ***S***_tip_〉. Diagonalization of ${\hat{H}}_{0}$ yields eigenstates, leading to four available ESR transitions of the Ti spin corresponding to four distinct combinations of the Fe and tip's spin states, |⇑〉_Fe_|⇑〉_tip_, |⇓〉_Fe_|⇑〉_tip_, |⇑〉_Fe_|⇓〉_tip_, and |⇓〉_Fe_|⇓〉_tip_. This dependence of the eigenstates on the tip spin is responsible for the periodic evolution of the resonance frequencies with the field angle by 180^o^, as observed from the pair of 0.72 nm (red markers in Figure 4a; and Figure S15, Supporting Information). An intuitive feature of ${\tilde{B}}_{0}$ is found in the case when the tip is far enough from Ti to ignore the Ti‐tip interaction (*J*_Ti,tip_ ≪ *J*_Ti,Fe_) so that the splitting becomes symmetric at about *θ*_ext_ = ±90^o^. An example can be seen in the data from the pair of 0.59 nm (blue markers in Figure 4), which were measured at a tunnel conductance of 0.05 nS (*V*_DC_ = 200 mV, *I*_DC_ = 10 pA) (Figure S16, Supporting Information).

Using the dependences of the eigenenergies of ${\hat{H}}_{0}$ on the field‐angle (*θ*_ext_) and tuning *J*_Ti,Fe_, we fit the resonance frequencies and peak splitting (Figure 4a,b), which excellently reproduced the detailed experimental features (solid curves in Figure 4a,b). The asymmetry in the resonance frequencies about *θ*_ext_ = +90^o^ and −90^o^ observed from the pair of 0.72 nm is revealed to stem from a sizable contribution of the tip spin at the relatively small tip‐Ti distance (red markers; *V*_DC_ = 30 mV, *I*_DC_ = 20 pA).

By considering both Ti‐tip and Ti‐Fe interactions, which contribute to the coherent RF driving fields ***B***_1,tip_ and ***B***_1,Fe_, respectively, the total driving field becomes ***B***_1_ = ***B***_1,tip_ + ***B***_1,Fe_, as illustrated in Figure 3c. Only the component of ***B***_1_ perpendicular to the total static field (***B***_0_), ***B***_1⊥_, contributes to the ESR of the Ti spin (*S* = 1/2), such that the Rabi rate is described by the following equation.

(3) $$\Omega \;=g\mu_{B}m_{S}\left|B_{1,\mathrm{tip}⊥}+B_{1,\mathrm{Fe}⊥}\right|/\hbar$$

with the magnetic quantum number of the Ti spin (*m*_S_ = 1/2). For some tips, such as the one shown in Figure 3, the two driving fields are either parallel (*ϕ* = 0) or antiparallel (*ϕ* = 180°), corresponding to the |⇑〉 or |⇓〉 states of the Fe, respectively (insets of Figure 3b). For *ϕ* = 180°, the net driving field (***B***_1⊥_) goes to zero at a critical Ti‐tip distance so that the ESR peak vanishes, and then it reappears with the opposite sign, resulting in the opposite Rabi rotation of the Bloch vector with a reappearance of the ESR signal (Figure 3d). For this special case of two driving fields, the Rabi rate of the Ti spin in Equation (3) can be rewritten as a simple sum of two contributions Ω_±_ = |Ω_tip_ ± Ω_Fe_|, where the + (−) sign represents the parallel (antiparallel) case. This model using Ω_±_, excellently reproduced the dependence of the ESR peak heights on the tunnel conductance (Supporting Information; solid curves in Figure 3b). The extrapolation of the peak heights to zero conductance yields finite intercepts of a peak height ratio of 0.64, which is largely determined by the thermal occupations of the two Fe states at the measurement temperature of 1.2 K.

In the following, we show quantification of the Fe‐induced driving field according to a model for ESR of a single spin on the surface, where the RF piezoelectric response Δ*Z*_1_(*t*) of the atom converts the RF electric field ***E*** (*t*) into a time‐varying magnetic field ***B***_1_(*t*) at the position of the atom.^[^ ^11^, ^12^ ^]^ Similar to ESR contributed from the Ti‐tip interaction, the Ti‐Fe interaction can play the same role when coupled with the RF voltage *V*_RF_(*t*) applied between the tip and substrate (Supporting Information). The Rabi frequency contributed from the Fe takes the form of the following equation.

(4) $$\Omega_{\mathrm{Fe}}=\left(\partial J_{\mathrm{Ti},\mathrm{Fe}}/\partial z\right)\cdot \Delta Z_{1}\cdot \sin\theta_{\mathrm{Ti}}$$

where *θ*_Ti_ is the polar angle of the Ti spin (Figure S19, Supporting Information). We assume an isotropic Ti‐Fe interaction with a decay length *d*_ex_ = 86 pm (Supporting Information) and take an adiabatic approximation to the RF displacement Δ*Z*_1_(*t*) in our frequency range of 20–30 GHz due to the bonding strengths of approximately few THz for both atoms to MgO. Here piezoelectric responses of both Ti and Fe atoms should be taken into account, such that the displacements of Ti by both static and RF electric fields are measured relative to those of Fe. Using *J*_Ti,Fe_ = 6.68 GHz for the pair of 0.59 nm as obtained in this work (Figure 4b and Figure S1, Supporting Information), Equation (4) yields Δ*Z*_1_/*V*_RF_ ≈ 0.20 pm mV^−1^ as the RF displacement for the Rabi rate Ω_Fe_/2*π* ≈ 25 MHz as measured when the tip's contribution was negligible (Figure 2c; *V*_DC_ = 100 mV, *I*_DC_ = 10 pA). This is comparable to the value (≈0.29 pm mV^−1^) derived for the ESR of an isolated Ti atom using the same model in a previous report.^[^ ^12^ ^]^ By considering piezoelectric motions of the two atoms of similar magnitudes but in opposite phases, we obtain Δ*Z*_1Ti_/*V*_RF_ ≈ Δ*Z*_1,Fe_/*V*_RF_ ≈ 0.10 pm mV^−1^. However, this is larger by two orders of magnitude than a theoretical estimation reported earlier^[^ ^7^ ^]^ when considering only the stretching of the atom‐MgO bonds as the sources of Δ*Z*_1_. The piezoelectric motion of the MgO layer and non‐linear piezoelectric responses of the atoms could contribute to such a large Δ*Z*_1_, as suggested in the previous works.^[^ ^7^ ^]^ Another possible contribution is from Ti‐Fe interactions through the substrate. This could be caused by the local distortion of the lattice at the adsorption sites of the atomic pairs of the 2–3 lattice‐site distance, which is close enough to lead to a considerable change in the local electronic properties and possibly opens an additional channel of spin‐spin interaction through the underlying lattice. Detailed DFT calculations would be needed to quantitatively answer what those relaxations are and how significant their impact is.

## 4 Conclusion

Utilizing a single‐atom magnet for ESR driving liberates on‐surface spins, which have been limited to a single qubit in the conventional ESR‐STM configuration. Furthermore, this approach enables the separation of driving and detection of ESR, providing longer relaxation and quantum coherence times, as shown in ref. [14]. Importantly, it enables atomic and molecular spins on surfaces as a platform for multi‐qubit operations. In this work, we investigated the electron spin resonance of a single Ti atom on MgO with Fe atoms in close proximity, unraveling the underlying driving mechanism of ESR in such a spin, exchange‐coupled to a single‐atom magnet. Together with atom manipulation techniques and the availability of even better single‐atom magnets,^[^ ^24^, ^25^, ^26^ ^]^ our work sheds light on an atomically precise design of on‐surface multi‐spin qubit structures.

## 5 Experimental Section

### Sample Preparation

An atomically clean Ag(100) substrate was prepared by alternating Ar ion sputtering and annealing cycles. MgO films were grown on the Ag substrate at 580 K by evaporating Mg in an O_2_ atmosphere of 1.1 × 10^−6^ Torr. Then, Fe and Ti atoms were deposited on the pre‐cooled MgO surface. All measurements were performed on Ti atoms bound to a bridge site of the MgO surface (that is, in the middle of two oxygen sites). Before measurements, the STM tip which was made of Pt/It wire was poked into the Ag(001) surface until satisfactory topographic and spectroscopic features were observed on atoms on MgO, after which Fe atoms were picked up by the STM tip from MgO (by applying a DC voltage pulse of 0.3 V) to create a spin‐polarized tip. The tip's spin polarization was calibrated with the asymmetry around zero bias in the d*I*/d*V* spectra of Ti on MgO.

### Measurements

Electron spin resonance (ESR) was performed in two ultrahigh‐vacuum (< 10^−10^ mbar) scanning tunneling microscopes (STMs). The data presented in Figures 1, 2, 3, 4, 5 were measured in a home‐built ^3^He‐cooled STM at *T* = 1.2 K equipped with a single‐axis superconducting magnet. The data presented in Figure 4 and Figures S14–S16, Supporting Information were measured in a commercial ^3^He‐cooled STM (Unisoku, USM1300) at *T* = 0.4 K equipped with a two‐axis superconducting magnet. High‐frequency transmission cables were installed on the STM systems as described in detail elsewhere.^[^ ^27^ ^]^ Continuous wave ESR spectra were acquired by sweeping the frequency of an RF voltage *V*_RF_ generated by an RF generator (Agilent E8257D) across the tunneling junction and monitoring changes in the tunneling current. For pulsed ESR, the RF generator was gated by the pulse outputs programmed in arbitrary waveform generators (Tektronix 7122C for 1K‐system; Tektronix 5000 for 0.4‐K system). The output signal of the RF generator was combined with a DC bias voltage through a bias tee (SigaTek, SB15D2). In both CW‐ and pulsed‐ESR measurements, the RF signals were chopped at 95 Hz and sent to a lock‐in amplifier (Stanford Research Systems SR860) and recorded by a DAQ module (National Instruments 6363). The bias voltage *V*_DC_ refers to the sample voltage relative to the tip. The STM constant‐current feedback loop was set to a low gain during the measurements.

## Conflict of Interest

The authors declare no conflict of interest.

## Author Contributions

S.P. and H.T.B. contributed equally to this work. C.P.L., S.P., and A.J.H. conceived the experiment. S.H., H.T.B., Y.W., K.Y., and C.P.L. performed experiments and data analysis. A.F. and J.F‐R. developed the analytical model for spin‐spin interaction. J.R‐G., C.W., and H.T.B. carried out numerical simulations and data fitting. All authors discussed the results and prepared the manuscript.

## Supporting information

Supporting Information

## Data Availability

The data that support the finding of this study are available in the main text and supplementary material of this article.
