# Supplementary material for: Electric‐Field‐Driven Spin Resonance by On‐Surface Exchange Coupling to a Single‐Atom Magnet
Source: Adv Sci (Weinh). 2023 Jul 19;10(27):2302033. doi: 10.1002/advs.202302033 (PMC10520627; doi:10.1002/advs.202302033)
Supplement: Supplementary file 1 — Supporting Information [file ADVS-10-2302033-s001.pdf]

ADVANCED SCIENCE
Open Access

Supporting Information

for *Adv. Sci.*, DOI 10.1002/advs.202302033

Electric-Field-Driven Spin Resonance by On-Surface Exchange Coupling to a Single-Atom Magnet

Soo-hyon Phark, Hong Thi Bui, Alejandro Ferrón, Joaquin Fernández-Rossier, Jose Reina-Gálvez, Christoph Wolf, Yu Wang, Kai Yang, Andreas J. Heinrich and Christopher P. Lutz**

Supporting Information for

Electric-field-driven spin resonance by exchange coupling to a single-atom magnet

Soo-hyon Phark, Hong Thi Bui, Alejandro Ferrón, Joaquin Fernández-Rossier, Jose Reina-Gálvez, Christoph Wolf, Yu Wang, Kai Yang, Andreas J. Heinrich, Christopher P. Lutz**

*Corresponding authors. Email: heinrich.andreas@qns.science, cplutz@us.ibm.com

This PDF file includes:

Supplementary Text
Figure S1 to S20

Supplementary Text

Section 1: ESR peak splitting measured on Ti-Fe pairs

1.1. Dependence on Ti-Fe separation

As discussed in the main text (Figure 1e), the ESR peak splitting measured on a Ti-Fe pair monotonically increased for a decreasing separation between two atoms. Figure S1a and b show ESR spectra measured on Ti-Fe pairs of five different separations and peak splitting with respect to the Ti-Fe separation. Assuming an isotropic exchange interaction, we applied an exponential dependence of splitting (Δf) on Ti-Fe separation ($r_{\mathrm{Ti,Fe}}$)[16,23]

$$\Delta f\left(r_{\mathrm{Ti,Fe}}\right) = J_0 \exp\left[-(r_{\mathrm{Ti,Fe}} - r_0)/d_{\mathrm{ex}}\right], \tag{S1}$$

where J_0 and d_{ex} are the exchange coupling energy at $r_{\mathrm{Ti,Fe}} = r_0$ and the decay length of the Ti-Fe interaction. Fitting the data in Figure S1b to Equation S1 with $r_0 = 0.59\ \mathrm{nm}$ resulted in $d_{\mathrm{ex}} = 86 \pm 27\ \mathrm{pm}$ and $J_0 = 3.22 \pm 0.45\ \mathrm{GHz}$ (red curve).

1.2. Dependence on pair orientations

We note that the pairs with a separation of 0.72 nm showed a mean splitting of 0.9 GHz but with a large dispersion of about 0.5 GHz (Figure S1). Due to the out-of-plane MAE of the Fe spin, a possible contribution from dipole-dipole interaction should have the same form as that from exchange interaction, leading to a total spin-spin interaction to be a simply combined shape $(J - D)\, \boldsymbol{S}_{\mathrm{Ti}} \cdot \boldsymbol{S}_{\mathrm{Fe}}$, where D is the dipole-dipole coupling strength for a given Ti-Fe separation. Thus, we expect that the total spin-spin interaction should be isotropic for the azimuthal orientation of a pair. To survey this, we measured the ESR peak splitting on Ti-Fe pairs of 0.72 nm with different pair orientations as shown in Figure S2. The splitting is anisotropic and doesn't show any regular dependence on the pair orientation. We speculate that this is from the difference in local environment of each pair and/or existence of defects nearby. This can induce local distortions in the position of the atoms as well as the underlying lattice, possibly leading to some spin-spin interactions not only via vacuum but also through the substrate. However, these issues are unresolvable using normal STM microscopy and spectroscopy due to the STM's resolution, which neither be able to see distortions smaller than ~10 pm around the atomic positions nor detect relaxations in the substrate below.

1.3. Dependence on tips

One possible origin of such a large dispersion of peak splitting observed from pairs of 0.72 nm is the anisotropic local field from the tip spin since it contributes an additional Zeeman energy to the atomic spins, which is comparable in size to the spin-spin interactions in the pair. We performed ESR on four pairs ('1' – '4') indicated in Figure S2 with 21 different tips and show the measured splitting in Figure S3. The mean splitting of the four pairs showed a large variation of 0.3–1.3 GHz as that shown in Figure S2 for the same pairs. However, the tip-dependent variation of the splitting on each pair is in the order of 0.1 GHz, indicating that the

ESR splitting was mainly contributed by the Ti-Fe interaction inherent in each pair when it formed on the surface.

Section 2: Eigenstates and eigenvalues of Ti-Fe pairs and Fe-Ti-Fe complex

2.1. Ti-Fe pair

To model the spin eigenstates of a Ti-Fe pair, we set up a Hamiltonian of the spin pairs

$$\hat{H}_{\text{pair}} = D_{\text{Fe}}S_Z^2 + C_{\text{Fe}}(S_+^4 + S_-^4) + J_{\text{Ti,Fe}}\boldsymbol{S}_{\text{Ti}} \cdot \boldsymbol{S}_{\text{Fe}} - g_{\text{Ti}}\mu_{\text{B}}\boldsymbol{S}_{\text{Ti}} \cdot \boldsymbol{B}_{\text{ext}} - g_{\text{Fe}}\mu_{\text{B}}\boldsymbol{S}_{\text{Fe}} \cdot \boldsymbol{B}_{\text{ext}}, \quad \text{(S2)}$$

composed of out-of-plane MAE of Fe $D_{\text{Fe}} = -4.7\ \text{meV}$,[6,20] four-fold in-plane anisotropy of Fe $C_{\text{Fe}} = 41\ \text{neV}$,[21] Zeeman terms for Ti and Fe spins with $B_{\text{ext,x}} = 0.891\ \text{T}$ and $B_{\text{ext,z}} = 0.125\ \text{T}$, and Ti-Fe exchange term with the coupling $J_{\text{Ti,Fe}}$ ranging 0–80 μeV, which corresponds to the peak splitting of 0–20 GHz observed from the ESR spectra (see Figure 4). g_{Ti} and μ_{B} are the g-factor of the Ti atom and Bohr magneton, respectively. The Hamiltonian is almost diagonal in the basis set $|m_{\text{Ti}}, m_{\text{Fe}}\rangle$ (Figure S4a), where m_{Ti} and m_{Fe} are magnetic quantum numbers of Ti and Fe spins, respectively, along the surface normal (**z** direction as depicted in Figure 1a). By diagonalizing the Hamiltonian, we found the four lowest eigenstates of the pair to be linear combinations of $|+1/2, +2\rangle$, $|-1/2, +2\rangle$, $|+1/2, -2\rangle$, and $|-1/2, -2\rangle$, whose eigenenergies are lower by ~ 13 meV than the other 6 eigenstates (Figure S4b). Together with the long spin relaxation time,[21,22] this allow us to simplify the analysis by treating the Fe as an Ising spin, and removing the MAE and four-fold in-plane anisotropy terms from the Hamiltonian. Each ESR peak originates from the resonance of the Ti spin between its two eigenstates $|+\rangle$ and $|-\rangle$ along the total static field $\boldsymbol{B}_0$ (see Figure 3c),[16] and each peak in the spectra of the Ti-Fe pairs corresponds to a quasi-static Fe spin states, $|\Uparrow\rangle$ and $|\Downarrow\rangle$, as denoted in Figure 1e.

2.2. Fe-Ti-Fe spin complex

To survey the eigenstates and possible ESR transitions of the Ti spin in a Fe-Ti-Fe pair shown in Figure 5, we set up a model Hamiltonian

$$\begin{aligned}\hat{H}_{\text{FTF}} = D_{\text{Fe}}S_{1Z}^2 + C_{\text{Fe}}(S_{1+}^4 + S_{1-}^4) + D_{\text{Fe}}S_{2Z}^2 + C_{\text{Fe}}(S_{2+}^4 + S_{2-}^4) - g_{\text{Fe}}\mu_{\text{B}}(\boldsymbol{S}_1 + \boldsymbol{S}_2) \cdot \boldsymbol{B}_{\text{ext}} \\ + J_{\text{Ti,Fe1}}\boldsymbol{S}_{\text{Ti}} \cdot \boldsymbol{S}_1 + J_{\text{Ti,Fe2}}\boldsymbol{S}_{\text{Ti}} \cdot \boldsymbol{S}_2 - g_{\text{Ti}}\mu_{\text{B}}\boldsymbol{S}_{\text{Ti}} \cdot \boldsymbol{B}_{\text{ext}}, \quad \text{(S3)}\end{aligned}$$

where two Fe spins, $\boldsymbol{S}_1$ and $\boldsymbol{S}_2$, are identical with two exchange couplings, $J_{\text{Ti,Fe1}}$ and $J_{\text{Ti,Fe2}}$, with the Ti spins, respectively. We used the same D_{Fe}, C_{Fe}, and g_{Fe} as those in the Hamiltonian for a Ti-Fe pair (Equation 1) and fixed $J_{\text{Ti,Fe1}}$ to be $J_{\text{Ti,Fe}} = 1.1\ \text{GHz}$ as extracted from Figure 4. We used $J_{\text{Ti,Fe2}}$ as a parameter to study a dependence of possible ESR transitions on the difference in the two Ti-Fe couplings. Similar to the case that a single Fe is coupled with the Ti spin, the Hamiltonian is almost diagonal in the basis set of the Zeeman product states of the three spins, $|m_{\text{Ti}}, m_{\text{Fe1}}, m_{\text{Fe2}}\rangle$ (Figure S5a). The eigenstates of $\hat{H}_{\text{FTF}}$ for

the lowest 8 eigenenergies are well decoupled by the four distinct spin orientations of two Fe spins, $|\Uparrow\Uparrow\rangle$, $|\Downarrow\Uparrow\rangle$, $|\Uparrow\Downarrow\rangle$, and $|\Downarrow\Downarrow\rangle$, as shown in Figure S5b.

2.3. The Ising character of the Fe spins in this work

Figure S4 and S5 clearly show the breakdown of the quantum states of the Fe in the coupled Fe-Ti spin systems. Each of 4 lowest eigenstates of the Ti-Fe spin pair are doublet overlaps only with the +2/-2 states by a large amount, which so to say "almost" when the out-of-plane field lifts the degeneracy of the two states in the zero-field splitting. We think that the Ising nature of the Fe is quite robust – it is far from being able to participate in quantum fluctuations because its anisotropy energy is so large (~20 meV, as found in Reference 20), and its Zeeman energy is detuned far from those of the Ti (~17 GHz at the external field used for Figure 1 and 2 in this work; see Reference 3). Our measurements and analysis indicate that the Fe spin's lifetime remains very long compared to the other timescales important for ESR and for maintaining coherence of the Ti: the Fe's spin fluctuates between its up and down states on a few tens of microsecond timescale (Reference 22), while the decoherence and relaxation of the Ti's spin occurs in a few hundreds of nanosecond at present (Reference 15).

Section 3: Model simulations of ESR transitions in Ti-Fe pairs

To survey the influence of the Ti-Fe separation on the resonance frequency of the Ti spin, we consider a simple picture that the Fe spin contributes an exchange field $\boldsymbol{B}_{\mathrm{Fe}}$ at the Ti position, as sketched in Figure 4c. Here we use $\widehat{H}_{\mathrm{pair}}$ (Equation S2) after removing the MAE (D_{Fe}) and four-fold in-plane anisotropy (C_{Fe}) terms according to the discussion in the Section 2.1. The Ti-Fe interaction $J_{\mathrm{Ti,Fe}}\boldsymbol{S}_{\mathrm{Ti}} \cdot \boldsymbol{S}_{\mathrm{Fe}}$ provides an additional Zeeman energy to the Ti spin

$$\boldsymbol{B}_{\mathrm{Fe}} = (J_{\mathrm{Ti,Fe}}/\mu_{\mathrm{B}})\boldsymbol{g}_{\mathrm{Ti}}^{-1} \cdot \boldsymbol{S}_{\mathrm{Fe}}, \quad \text{(S4)}$$

where $\boldsymbol{g}_{\mathrm{Ti}}$ is the g-tensor of the Ti spin. Considering the Fe spin as a classical magnetic dipole pointing along the $\pm\hat{\boldsymbol{z}}$ direction, we take an approximation of $\boldsymbol{S}_{\mathrm{Fe}} = \langle S_{\mathrm{Fe,z}}\rangle$. This simplifies the Equation S4 such that $\boldsymbol{B}_{\mathrm{Fe}} = \pm J_{\mathrm{Ti,Fe}}\langle S_{\mathrm{Fe,z}}\rangle/\mu_{\mathrm{B}} g_{\mathrm{Ti,z}}\,\hat{\boldsymbol{z}}$, as denoted in Figure 4c by $\boldsymbol{B}_{\mathrm{Fe},\Uparrow}$ and $\boldsymbol{B}_{\mathrm{Fe},\Downarrow}$ for the Fe's $|\Uparrow\rangle$ and $|\Downarrow\rangle$ states, respectively. Diagonalization of $\widehat{H}_{\mathrm{pair}}$ (Equation S2) yields four eigenstates, leading to two available ESR transitions, $f_{\Uparrow}$ and $f_{\Downarrow}$, of the Ti spin corresponding to two distinct Fe spin states, which determine peak splitting $\Delta f = f_{\Uparrow} - f_{\Downarrow}$. We present dependences of $f_{\Uparrow}$, $f_{\Downarrow}$, and Δf on Ti-Fe separation in Figure S6. Note the blue shifts of the resonance frequencies for a decreasing Ti-Fe separation, as discussed in the main text.

Section 4: Analysis of Rabi rates vs. tunnel conductance in Figure 2c

4.1. Two driving fields

The Rabi rate (Ω) of the Ti spin is directly proportional to the magnitude of the effective total driving field $\boldsymbol{B}_{1\perp}$ (see Figure 3c), residing in the plane perpendicular to the total static field $\boldsymbol{B}_0$,

$$\Omega = \alpha B_{1\perp} \tag{S5}$$

with a proportionality constant α. The $\boldsymbol{B}_{1\perp}$ is the vector sum of the tip-contributed ($\boldsymbol{B}_{1,\mathrm{tip}\perp}$) and Fe-contributed ($\boldsymbol{B}_{1,\mathrm{Fe}\perp}$) ones as depicted in Figure 3c, hence its magnitude is calculated by

$$B_{1\perp} = \sqrt{B_{1,\mathrm{Fe}\perp}^2 + B_{1,\mathrm{tip}\perp}^2 + 2B_{1,\mathrm{Fe}\perp}B_{1,\mathrm{tip}\perp}\cos\phi}, \tag{S6}$$

where ϕ is the angle between the two vectors $\boldsymbol{B}_{1,\mathrm{tip}\perp}$ and $\boldsymbol{B}_{1,\mathrm{Fe}\perp}$. By assuming that the contribution from Fe ($\boldsymbol{B}_{1,\mathrm{Fe}\perp}$) for a given Ti-Fe separation is independent of the tunnel conductance (σ_{tun}), i.e. tip-Ti distance ($d_{\mathrm{tip,Ti}}$), we show in Figure S8b the magnitude of the total driving field ($B_{1\perp}$) as a function of that of the tip-originated driving field $B_{1\mathrm{tip}\perp}$. First, we note two special cases: (i) with $\phi = 0$ ($\boldsymbol{B}_{1,\mathrm{tip}\perp}$ and $\boldsymbol{B}_{1,\mathrm{Fe}\perp}$ are parallel.), $B_{1\perp}$, and thus Ω, is maximum for the whole range of $B_{1,\mathrm{tip}\perp}$. (ii) with $\phi = 180°$ ($\boldsymbol{B}_{1,\mathrm{tip}\perp}$ and $\boldsymbol{B}_{1,\mathrm{Fe}\perp}$ are antiparallel.), $B_{1\perp}$ becomes zero at $B_{1,\mathrm{tip}\perp} = B_{1,\mathrm{Fe}\perp}$ and bounces back for $B_{1,\mathrm{tip}\perp} < B_{1,\mathrm{Fe}\perp}$. Both cases show saturation of $B_{1\perp}$ to $B_{1,\mathrm{Fe}\perp}$ at $B_{1,\mathrm{tip}\perp} = 0$, which is trivial. These two cases lead to the tunnel conductance dependence of the ESR peak amplitudes shown in Figure 3b. For an arbitrary choice of two driving vectors, neither parallel nor antiparallel to each other, $B_{1\perp}$ shows a trend in between the two cases mentioned above and also saturates to the same point at $B_{1,\mathrm{tip}\perp} = 0$.

4.2. Fit of the Rabi rates vs. tunnel conductance in Figure 2c

Using the linear dependence of the Rabi rate of an isolated Ti spin on the tunnel conductance (gray in Figure 2c), we can scale the conductance axis to the Rabi rate contributed only by tip (Ω_{tip}), as discussed in the following. The tunnel conductance σ_{tun} and tip-originated driving field $B_{1,\mathrm{tip}\perp}$ can be described using the tip height (z) as follows (*11*):

$$\sigma_{\mathrm{tun}} = \sigma_0\, e^{-z/d_0}, \tag{S7}$$

$$B_{1,\mathrm{tip}\perp} = c_{1,\mathrm{tip}\perp}\, e^{-z/d_{\mathrm{ex}}}/z, \tag{S8}$$

where σ_0, d_0, and d_{ex} are the tunnel conductance at the point contact, decay lengths of tunneling probability, and that of exchange coupling between tip and Ti spins. Here, we take the tip height to be zero at the tip-Ti point contact. Using (S7), (S8) is transformed into

$$B_{1,\mathrm{tip}\perp} = \frac{c_{1,\mathrm{tip}\perp}}{d_0}\left(\frac{\sigma_{\mathrm{tun}}}{\sigma_0}\right)^{d_0/d_{\mathrm{ex}}}\frac{1}{\ln(\sigma_0/\sigma_{\mathrm{tun}})}. \tag{S9}$$

Set the parts of the tunnel conductance in (S7) as x_{tip} and $c_{1,\mathrm{tip}\perp}/d_0$ as β, together with the relation in (S5), we obtain

$$\Omega_{\text{tip}}/\alpha\beta = x_{\text{tip}},\ \text{where}\ x_{\text{tip}} \equiv \left(\frac{\sigma_{\text{tun}}}{\sigma_0}\right)^{d_0/d_{\text{ex}}} \frac{1}{\ln(\sigma_0/\sigma_{\text{tun}})}. \tag{S10}$$

Then using (S6) and (S10), the Rabi rate (Ω; y-axis of Figure 2c) is scaled into

$$\Omega/\alpha\beta = \left(x_{\text{Fe}}{}^2 + x_{\text{tip}}{}^2 + 2\, x_{\text{Fe}} x_{\text{tip}} \cos\phi\right)^{1/2} = y, \tag{S11}$$

where $x_{\text{Fe}} \equiv B_{1,\text{Fe}\perp}/\beta$. The plots $\Omega(\sigma_{\text{tun}})$ in Figure 2c can be transformed into $y(x_{\text{tip}})$ using (S10) and (S11), and $\alpha\beta$ can be extracted from the data for an isolated Ti using the relation in (S9).

We obtained $d_0 = 43.4$ pm by using Equation S7 with $\sigma_0 = 0.6$ mS from the point contact experiment data on a bridge-site Ti atom.[23] The d_{ex} was extracted using a tunnel conductance dependence of the ESR resonance frequency (f_{res}) measured on an isolated Ti, as shown in Figure S9a. The linear dependence of f_{res} on the tunnel conductance suggests the exchange interaction between tip and Ti as a dominant contribution to the shift of the resonance frequency. From a linear fit, we obtained f_{res} of 22.485 GHz at zero conductance and calculated the shift of f_{res} (Δf_{res}) at each tunnel conductance. Assuming an exponential dependence of Δf_{res} on the tip height (z)

$$\Delta f_{\text{res}}(z) \propto \exp[-(z - z_0)/d_{\text{ex}}], \tag{S12}$$

we obtained the decay length of $d_{\text{ex}} = 38.2$ pm for the Ti-tip interaction. Together with the d_0 and σ_0 from above, we scaled the $\Omega(\sigma_{\text{tun}})$ plots in Figure 2c into the $y(x_{\text{tip}})$ plane as shown in Figure S10. We fit the data using (S11) with x_{Fe} and ϕ as fitting parameters, resulting in the zero conductance Rabi rates Ω_0 for the two pairs of 0.59 and 0.72 nm.

Section 5: Coherence of Ti spin in the Ti-Fe pair

The coherence times are limited mainly by three known sources: (i) the presence of the magnetic STM tip and its unintended vibrations, (ii) the tunneling electrons that pass between tip and surface, and (iii) the electrons that tunnel from either the tip or surface electrode, scatter from the atom, and return to the original electrode. The technique studied here is applicable to removing decoherence due to the first two – the tip-dependent vibration and tunneling. In Reference 15, we developed a way to control the atomic spins "remote" from the tip and demonstrated multi-spin measurements using a single tip. We obtained much longer relaxation ($T_1 \sim 150$ ns) and coherence ($T_2 \sim 300$ ns) times of a remote spin, which is expected to be free from the tip-vibration and tunneling current. The in-depth technical exploration of the driving mechanism presented here continues to use a tip still positioned over the spin-resonant atom, so coherence times are expected to improve significantly here only in the special case of very small tunneling current, where we show that the Rabi rate continues to be considerable even though the limit of small current is approached. We did not see a considerable enhancement of spin coherence in the Rabi oscillation data within the tunneling conductance regime of this work (down to 0.05 nS) as shown in Figure S11a, where

the tip-vibration-induced decoherence might be still dominant. Instead, we show here coherence time (T_2^{echo}) measured on a Ti with Fe located in 0.6 nm apart using the spin-echo scheme (see Figure S11b), by which we believe to effectively remove the tip-vibration-induced decoherence. The T_2^{echo} showed a dependence on tunneling current, extrapolating to 230 ns at zero current, which is comparable to the previous result from Ti atoms on MgO surface (Reference 3 and 4). This is an order longer than the coherence times extracted from decay of Rabi oscillations (Figure S11a) and further proves that the coherence of Ti spin in this spin pair is not notably affected by the slow fluctuation of the nearby Fe spin.

Section 6: Characterization of the tip used in Figure 4

6.1. Analysis of CW- and pulsed-ESR data of an isolated Ti spin

To have a quantitative insight on the contribution of the tip spin to the measurements of the Ti-Fe pairs (Figure 4), we performed both CW- and pulsed-ESR measurements on an isolated Ti with the same tip. In Figure S13, we show resonance frequency (f_{ESR}), Rabi rate, and CW-ESR amplitude as a function of the angle (θ_{ext}) of the external field. A time-independent Hamiltonian $\widehat{H}_0$ of this system can written as

$$\widehat{H}_0 = -g_{\mathrm{Ti}}\mu_{\mathrm{B}}\boldsymbol{S}_{\mathrm{Ti}} \cdot \boldsymbol{B}_{\mathrm{ext}} - g_{\mathrm{Fe}}\mu_{\mathrm{B}}\boldsymbol{S}_{\mathrm{tip}} \cdot \boldsymbol{B}_{\mathrm{ext}} + J_{\mathrm{Ti,tip}}\boldsymbol{S}_{\mathrm{Ti}} \cdot \boldsymbol{S}_{\mathrm{tip}}, \qquad \text{(S13)}$$

where the first two terms represent the Zeeman energies of the Ti and tip spins, and the last term does the interaction between two spins. We referred to the g-factor of the Ti spin as reported previously.[9] As discussed in the main text, the tip's spin used for the measurements in Figure 4 was well described, like the Fe spin in this work, by a classical magnetic moment of uniaxial anisotropy along a direction

$$\boldsymbol{n}_{\mathrm{tip}} = \left(\sin\theta_{\mathrm{tip}}\cos\phi_{\mathrm{tip}}, \sin\theta_{\mathrm{tip}}\sin\phi_{\mathrm{tip}}, \cos\theta_{\mathrm{tip}}\right), \qquad \text{(S14)}$$

where θ_{tip} and ϕ_{tip} are the polar and azimuthal angles of $\boldsymbol{n}_{\mathrm{tip}}$ in the Cartesian coordinates. We choose coordinates, where the sample surface and external field are confined in the xy- and xz-plane, respectively, so that the directions of the external field and Ti spin are described by conventional definitions of the polar and azimuthal angles, θ_{ext} and $\boldsymbol{n}_{\mathrm{tip}}(\theta_{\mathrm{tip}}, \phi_{\mathrm{tip}})$, as illustrated in Figure S15a. Since the Ti-tip interaction was smaller than the Zeeman energy of the Ti by about two orders of magnitude (Figure S16), we approximate the direction of the Ti spin to be along the external magnetic field. Hence, the external field and tip spin can be written as

$$\boldsymbol{B}_{\mathrm{ext}} = B_{\mathrm{ext}}\boldsymbol{n}_{\mathrm{ext}}, \text{ where } \boldsymbol{n}_{\mathrm{ext}} = \hat{\boldsymbol{x}}\sin\theta_{\mathrm{ext}} + \hat{\boldsymbol{z}}\cos\theta_{\mathrm{ext}}, \qquad \text{(S15)}$$

$$\boldsymbol{S}_{\mathrm{tip}} = \pm\left|\langle S_{\mathrm{tip}}\rangle\right|\boldsymbol{n}_{\mathrm{tip}}. \qquad \text{(S16)}$$

Diagonalization of the Hamiltonian (S13) results in four eigenstates $|+\rangle|\Uparrow\rangle$, $|-\rangle|\Uparrow\rangle$, $|+\rangle|\Downarrow\rangle$, $|-\rangle|\Downarrow\rangle$, where the first and second parts of each represent Ti and tip spin states, and corresponding eigenenergies parameterized by θ_{tip}, ϕ_{tip}, and $J_{\mathrm{Ti,tip}}\left|\langle S_{\mathrm{tip}}\rangle\right|$. We obtain two

possible ESR transitions of the Ti spin for two spin states of the tip ($|+\rangle|\Uparrow\rangle \leftrightarrow |-\rangle|\Uparrow\rangle$ and $|+\rangle|\Downarrow\rangle \leftrightarrow |-\rangle|\Downarrow\rangle$) as a function of θ_{ext}. A simulation of the resonance frequency using this model with $\theta_{\text{tip}} = 65°$ and $\phi_{\text{tip}} = 75°$ is in a great agreement with the experiment (red curves in Figure S15b). The overall θ_{ext}-dependent variation of the resonance frequency ranging about 2 GHz stemmed from the anisotropy in the g-factor of the Ti spin (g_{Ti}).[10] We note a crossing of two simulation curves at about $\theta_{\text{ext}} = -25°$, across which the Zeeman energy forces the tip's spin state to flip from $|\Uparrow\rangle$ to $|\Downarrow\rangle$ (or vice versa).

Time-dependent perturbation theory deduces the Rabi rate (Ω) of a spin $\boldsymbol{S}$ ($S = 1/2$)

$$\hbar\Omega = g\mu_{\text{B}}\langle + \,|\, \boldsymbol{B}_1 \cdot \boldsymbol{S} \,|\, - \rangle \tag{S17}$$

for a given time-varying magnetic field $\boldsymbol{B}_1$. Only the component of $\boldsymbol{B}_{1\text{tip}}$ perpendicular to the total static magnetic field $\boldsymbol{B}_0$ drives the ESR of the spin, leading to a dependence of the Rabi rate on the angle γ between $\boldsymbol{B}_0$ and $\boldsymbol{B}_{1\text{tip}}$

$$\Omega(\gamma) = \Omega_{\gamma=90°} \sin\gamma \ = \Omega_{\gamma=90°}\sqrt{1-\cos^2\gamma}. \tag{S18}$$

Here γ is approximately determined by the directional cosine between two unit vectors, $\boldsymbol{n}_{\text{ext}}$ and $\boldsymbol{n}_{\text{tip}}$, $\cos\gamma = \boldsymbol{n}_{\text{ext}} \cdot \boldsymbol{n}_{\text{tip}}$. The θ_{ext}-dependence of the Rabi rate is then described by

$$\Omega(\theta_{\text{ext}}) = \Omega_{\gamma=90°}\sqrt{1-(\sin\theta_{\text{tip}}\cos\phi_{\text{tip}}\sin\theta_{\text{ext}} + \cos\theta_{\text{tip}}\cos\theta_{\text{ext}})^2} \tag{S19}$$

with the polar and azimuthal angles of the tip ($\theta_{\text{tip}}, \phi_{\text{tip}}$). Note that the tip spin flips when γ changes across 90° or 270°, where the Rabi rate is the maximum ($\Omega_{\gamma=90°}$), leading to a continuous evolution of the Rabi rate with a periodicity of 180° in the θ_{ext} axis. With the θ_{tip} and ϕ_{tip} from the analysis of the resonance frequency (Figure S15b), we simulated the θ_{ext}-dependence of the Rabi rate as shown in Figure S15c (solid orange curve), which is in good agreement with the experimental data.

The influence of the uniaxial anisotropy of the tip spin also appears in the θ_{ext}-dependence of the ESR peak amplitude. We calculated a simulation curve (purple curve in Figure S15d) using the model introduced in a previous report with the anisotropy of our tip,[10] $\boldsymbol{n}_{\text{tip}}(\theta_{\text{tip}}, \phi_{\text{tip}})$, extracted from the analysis of the resonance frequency (Figure S15b). The result fits in great agreement with the experimental data, with assignments of critical angles where the ESR amplitude becomes minima when the spins of the tip and Ti are either parallel or perpendicular due to its dependence on the magnetoresistance of the tunnel junction. These angles for the perpendicular and parallel configurations of the two spins coincide with the angles for the maximum (Ω_{max}) and minimum (Ω_{min}) Rabi rates, a compelling evidence that the tip spin provides the driving field of the ESR on the Ti spin, which is maximum when it is perpendicular to the total static magnetic field.

6.2. CW- and pulsed-ESR data of an isolated Ti: tunnel conductance dependence

Figure S16A and B show CW- and pulsed-ESR data measured at four different tunnel conductance on the same isolated Ti atom and with the same tip used for the measurements in Figure 4 and Figure S15. In Figure S16c, we show the dependence of the resonance frequency on the tunnel conductance. From a linear fit, we extrapolated the resonance frequency at zero conductance and estimated the tip-field-induced Zeeman energy of 0.008 GHz at 0.05 nS. This is smaller by ~ 600 times than the Ti-Fe interaction ($J_{\mathrm{Ti,Fe}}$) of 4.91 GHz in the pair with a separation of 0.59 nm and at the field angle of 72°, as can be seen in the data in Figure 4 and Figure S14c where the same tunnel conductance (0.05 nS: V_{DC} = 200 mV, I_{DC} = 10 pA) was used. This fulfills the condition $J_{\mathrm{Ti,Fe}} \gg J_{\mathrm{Ti,tip}}$ in the discussion of the main text. In addition, only a noisy fluctuation was observed in the Rabi measurement at 0.1 nS (Figure S16b), which is also a compelling evidence, supporting the negligible contribution of the tip-induced field to the data in Figure S14c.

Section 7: ESR peak height and asymmetry vs. tunnel conductance in Figure 3

7.1. Peak height

The contribution of the Rabi rate Ω to the ESR peak height is described by $\Omega^2 T_1 T_2/(1+\Omega^2 T_1 T_2)$, where T_1 and T_2 are the energy relaxation time and dephasing time, respectively.[10,S1] Using a unitless Rabi rate $\Omega'_{\pm} \equiv \Omega_{\pm}\sqrt{T_1 T_2}$ as fitting parameters, our model using the Rabi rates given in Equation 6 excellently reproduced the tunnel conductance dependence of the ESR peak heights (solid curves in Figure 3b). This specific behavior was observed only with certain tips, providing a collinear alignment to the Fe spin's direction, while other tips showed a similar behavior but generally yielded incomplete cancellation (Figure S18).

7.2. Peak asymmetry

In ESR of an isolated single spin, the in-phase transverse component $\langle S_x \rangle$ of the steady-state spin expectation value is antisymmetric across the spin resonance in the frequency sweep (Reference S1). When the tip spin and target spin are neither parallel nor antiparallel (this is always true when ESR is detected, since otherwise the tip-induced driving field $\boldsymbol{B}_1$ collapses to zero; Reference 10), the tip spin component perpendicular to the quantization axis of the target spin homodyne detects $\langle S_x \rangle$ and gives rise to asymmetry of resonance peaks in continuous wave ESR-STM measurements.

For a Ti-Fe pair, we expect a dependence of peak asymmetry on tunnel conductance since the total driving field changes its direction as well as magnitude as a function of tip-Ti distance. The simplest case to clearly observe such a dependence is ESR of a pair, where two transverse driving fields $\boldsymbol{B}_{1,\mathrm{tip},\perp}$ and $\boldsymbol{B}_{1,\mathrm{Fe},\perp}$ are collinear as presented in Figure 3. We observed a reversal of the peak asymmetry across the tip-Ti distance, where the peak amplitude disappears (i.e., $\boldsymbol{B}_{1,\mathrm{tip},\perp}$ and $\boldsymbol{B}_{1,\mathrm{Fe},\perp}$ are antiparallel and completely cancel out) as shown in Figure S13b. This is

also a clear evidence that a reversal in the direction of the total transverse driving field $\boldsymbol{B}_{1,\perp}$ occurs, corresponding to phase shift of 180° and reversal of Rabi rotation across the critical tip-Ti distance, as depicted in Figure 3d. In contrast, measurements on an isolated Ti spin using the same tip showed peak asymmetry that is almost independent of the tunnel conductance (Figure S13a). This is consistent to our picture that only tip magnetic moment contributes to the driving field in this case, which changes only its magnitude but not direction as a function of tunnel conductance.

For the general case, in which $\boldsymbol{B}_{1,\text{tip},\perp}$ and $\boldsymbol{B}_{1,\text{Fe},\perp}$ are neither parallel nor antiparallel, the asymmetry of ESR peak would show a gradual change as the tip height is changed, which changes the magnitude of the tip-induced driving field ($\boldsymbol{B}_{1,\text{tip},\perp}$). Here, the phase shift (φ) is determined by the trigonometric relationship of the three vectors ($\boldsymbol{B}_{1,\text{tip},\perp}$, $\boldsymbol{B}_{1,\text{Fe},\perp}$, $\boldsymbol{B}_{1,\perp}$), as depicted in Figure S13c. In this work, the experiments shown in Figure 2 correspond to this case, and we extracted the angle between the transverse components of the two driving fields ($\boldsymbol{B}_{1,\text{tip},\perp}$ and $\boldsymbol{B}_{1,\text{Fe},\perp}$) from the analysis of the Rabi rates as a function of tunnel conductance (see Supplementary section 4.2 and Figure S10).

Section 8: A model for driving ESR of a Ti spin contributed from nearby Fe

8.1. Application of 'piezoelectric driving model' to the Rabi rate contributed from Fe

The Ti-Fe interaction $J_{\text{Ti,Fe}}$ generates an inhomogeneous local magnetic field $\boldsymbol{B}_{\text{Fe}}$ at the position of the Ti atom. Then, the Ti spin in the piezo-electric motion $\Delta Z_1(t)$, induced by $\boldsymbol{E}_{\text{RF}}(t)$, can feel an effective time-varying magnetic field $\boldsymbol{B}_{1,\text{Fe}}(t)$ which is able to drive its ESR. The first order time-dependent perturbation theory yields the Rabi rate, contributed from the Fe spin,

$$\hbar\Omega_{\text{Fe}} = g\mu_{\text{B}}\langle + | \boldsymbol{B}_{1,\text{Fe}} \cdot \boldsymbol{S}_{\text{Ti}} | - \rangle \tag{S20}$$

for the ESR transition between two eigenstates of the Ti spin, $|+\rangle$ and $|-\rangle$. For considering the electric field in the tunnel junction, we take a parallel capacitor geometry, composed of the tip and Ag substrate as two metallic electrodes, in which the atoms have piezoelectric responses to the electric field $\boldsymbol{E}(t) = \boldsymbol{E}_{\text{DC}} + \boldsymbol{E}_{\text{RF}}(t) \approx [V_{\text{DC}} + V_{\text{RF}}(t)]/d$, (Figure S19). Here the voltage drop in the junction occurs mostly in the vacuum region due to the larger dielectric constant of MgO than that of vacuum by the factor of ~10,[S2] such that we take $d \approx$ 0.5 nm, together with the tip-atom distance at the measurement tunnel condition when the tip's contribution was negligible (Figure 2c; V_{DC} = 100 mV, I_{DC} = 10 pA) as referred to.[21,23]

Following the discussion on so called the 'piezoelectric model',[11] the field induced by Fe $\boldsymbol{B}_{\text{Fe}}$ can generate a time-varying field with Equation S4,

$$\boldsymbol{B}_{1,\text{Fe}}(t) \approx (\partial B_{\text{Fe}}/\partial z) \cdot \Delta Z_1(t) = (\langle S_{\text{Fe,z}}\rangle/\mu_{\text{B}} g_{\text{Ti,z}}) \cdot (\partial J_{\text{Ti,Fe}}/\partial z) \cdot \Delta Z_1(t),$$

which leads to the Rabi rate contributed from the Fe spin (Equation S20):

$$\Omega_{\mathrm{Fe}} = (\partial J_{\mathrm{Ti,Fe}}/\partial z) \cdot \Delta Z_1 \cdot \sin\theta_{\mathrm{Ti}}. \tag{S21}$$

The sine factor of the polar angle of the Ti (θ_{Ti}) appears since only the component of $\boldsymbol{B}_{1,\mathrm{Fe}}(t)$ perpendicular to the total static field ($\boldsymbol{B}_0$; i.e. the direction of the Ti spin) contributes to the Rabi rate. On assuming an isotropic Ti-Fe interaction $J_{\mathrm{Ti,Fe}}$ (Equation 1), the Equation S21 becomes

$$\Omega_{\mathrm{Fe}} = -(J_{\mathrm{Ti,Fe}}/d_{\mathrm{ex}}) \cdot (\Delta Z_0/r) \cdot \Delta Z_1 \cdot \sin\theta_{\mathrm{Ti}}, \tag{S22}$$

where d_{ex}, r, and ΔZ_0 are decay length of J_{TiFe}, Ti-Fe separation, and static displacement of the Ti atom.

Since both Ti and Fe atoms are experiencing the electric field $\boldsymbol{E}_{\mathrm{RF}}(t)$ in this work, piezoelectric responses of both atoms should be taken account. Thus, both static (ΔZ_0) and RF (ΔZ_1) displacements should be measured relative to those of the Fe, such that $\Delta Z_0 = Z_{0,\mathrm{Ti}} - Z_{0,\mathrm{Fe}}$ and $\Delta Z_1 = Z_{1,\mathrm{Ti}} - Z_{1,\mathrm{Fe}}$, respectively. The static displacement ΔZ_0 is the sum of contributions from the atom-surface bonding ($\Delta Z_{0,\mathrm{eq}} = Z_{\mathrm{eq,Ti}} - Z_{\mathrm{eq,Fe}}$) and DC electric field ($\Delta Z_{0,\mathrm{DC}} = Z_{\mathrm{DC,Ti}} - Z_{\mathrm{DC,Fe}}$). The equilibrium displacement $\Delta Z_{0,\mathrm{eq}}$ of about 33 pm was found from our density functional theory (DFT) calculations. Resonance frequencies of atom-surface bonds for both Ti and Fe are expected to be in the range of ~ a few THz,[12] thus we take an adiabatic approximation to the piezoelectric displacements of both atoms in our RF electric field $\boldsymbol{E}_{\mathrm{RF}}(t)$ of a frequency range of 20–30 GHz, which allows a condition $\Delta Z_{0,\mathrm{DC}}/V_{\mathrm{DC}} = \Delta Z_1/V_{\mathrm{RF}}$.

8.2. Density functional theory calculations for the adsorption geometry of TiH and Fe

We performed density functional theory (DFT) calculations using Quantum Espresso 7.1[S3] to obtain the adsorption geometry of a hydrogenated Ti (TiH) and a Fe on 2 monolayer-thick MgO on Ag(100). Projector augmented wave pseudopotentials from the PSLibrary[S4] were used for all elements and the cutoff for the kinetic energy and charge density was set to 80 Ry and 800 Ry, respectively. Integration of the Brillouin zone was performed on a 3x3x1 regular grid of a slab-vacuum cell consisting of 4 monolayers of silver capped by 2 monolayers of magnesium oxide expanded into a 3x3 lateral cell and padded with 15 Ang of vacuum in the z-direction. Dispersive forces were treated using the Grimme-d3 method.[S5] To obtain the equilibrium geometry TiH (Fe) was added on a bridge (oxygen top) side and the whole system was relaxed until the residual forces were less than 0.01 eV/Ang. The equilibrium geometry for each system is shown in Figure S20.

[S1] F. Delgado, J. Fernández-Rossier, *Prog. Surf. Sci*. **2017**, *92*, 40–82.

[S2] M. A. Subramanian, R. D. Shannon, B. H. T. Chai, M. M. Abraham, M. C. Wintersgill, *Phys. Chem. Minerals* **1989**, *16*, 741−746.

[S3] P. Giannozzi, O. Andreussi, T. Brumme, O. Bunau, M. B. Nardelli, M. Calandra, R. Car, C. Cavazzoni, D. Ceresoli, M. Cococcioni, N. Colonna, I. Carnimeo, A. D. Corso, S. de Gironcoli, P. Delugas, R. A. DiStasio Jr, A. Ferretti, A. Floris, G. Fratesi, G. Fugallo, R. Gebauer, U. Gerstmann, F. Giustino, T. Gorni, J. Jia, M. Kawamura, H.-Y. Ko, A. Kokalj, E. Küçükbenli, M. Lazzeri, M. Marsili, N. Marzari, F. Mauri, N. L. Nguyen, H.-V. Nguyen, A. Otero-de-la-Roza, L. Paulatto, S. Poncé, D. Rocca, R. Sabatini, B. Santra, M. Schlipf, A. P. Seitsonen, A. Smogunov, I. Timrov, T. Thonhauser, P. Umari, N. Vast, X. Wu, S. Baroni, *J. Phys.*: *Condens. Matter* **2017**, *29*, 465901.

[S4] A. D. Corso, *Comp. Mater. Sci*. **2014**, *95*, 337−350.

[S5] S. Grimme, A. Hansen, J. G. Brandenburg, C. Bannwarth, *Chem. Rev*. **2016**, *116,* 5105–5154.

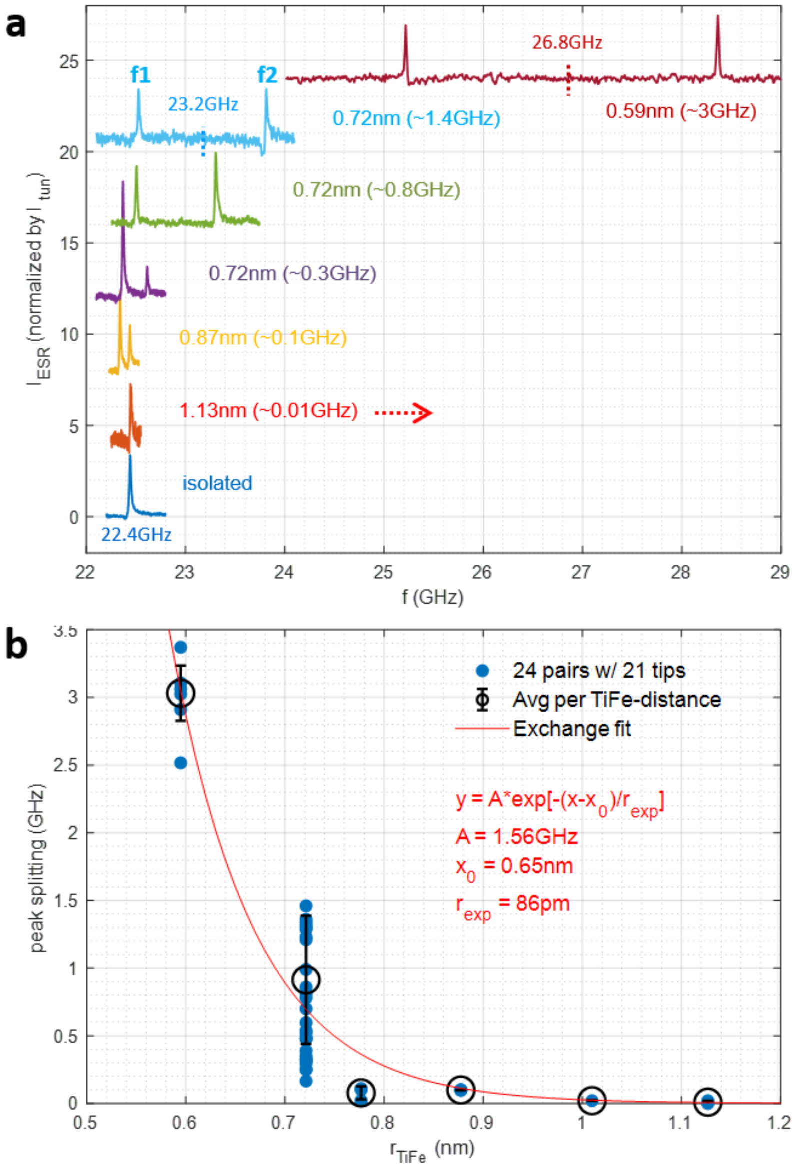

Figure S1. ESR peak splitting of pairs with various Ti-Fe separations. (a) Spectra measured on seven pairs of different Ti-Fe separations (V_{RF} = 20 mV, T = 1.2 K, B_{ext} = 0.9 T, θ_{ext} = 82°). The inset is a zoom-in of the spectrum on the pair with a separation of 1.13 nm. (b) Dependence of peak splitting on Ti-Fe separation, extracted from 24 pairs. The red solid curve is a fit to an exponentially decaying function in the Equation S1, resulting in the decay length d_{ex} of the Ti-Fe interaction shown in the table (red, inset), with those from the other spin pairs for comparison.[16,23]

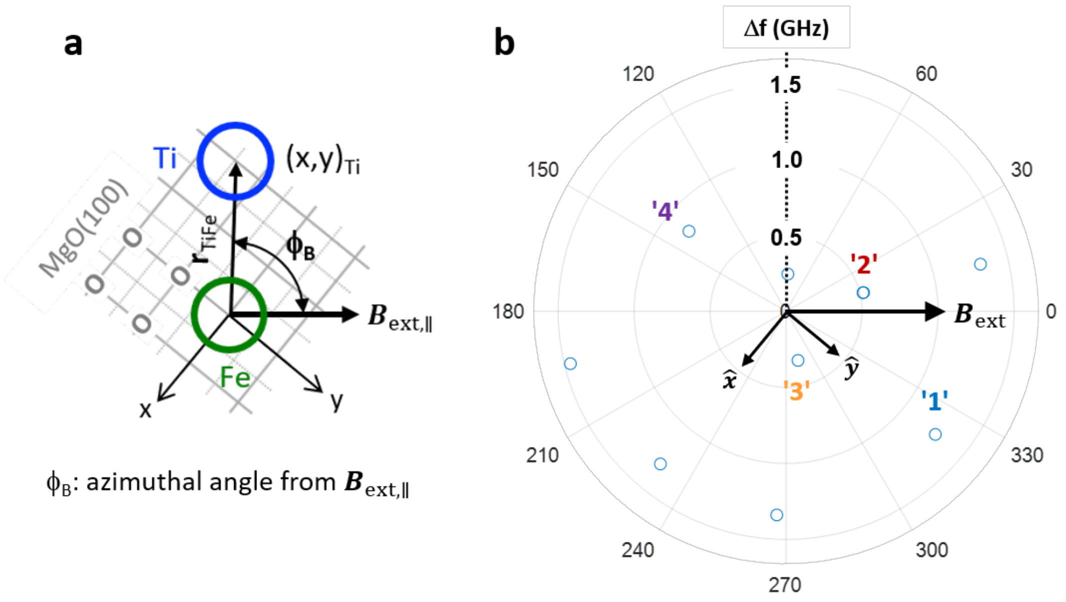

Figure S2. Dependence of ESR peak splitting on pair orientations. (a) A schematic illustrating a Ti-Fe pair with separation of 0.72 nm. The position of Ti is measured in the polar coordinate ($r_{\mathrm{Ti,Fe}}$, ϕ_{B}), measured from the position of Fe and in-plane component of the external field ($\boldsymbol{B}_{\mathrm{ext},\parallel}$). The gray mesh represents the underlaying MgO(100) lattice. (b) A collection of ESR peak splitting measured on pairs with separation of 0.72 nm as a function of azimuthal angle (ϕ_{B}) of Ti atom. The radius of the graph denotes the splitting (Δf) (B_{ext} = 0.9 T, θ_{ext} = 82°).

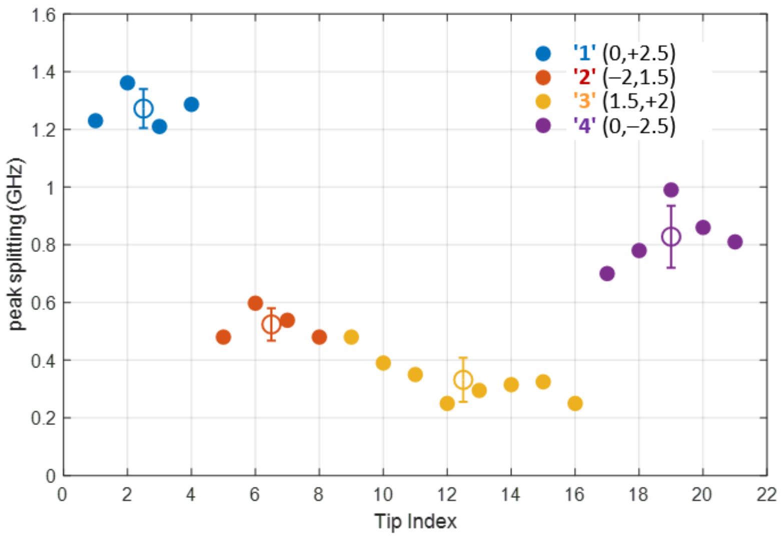

Figure S3. Dependence of ESR peak splitting on different tips used. ESR peak splitting measured from the four pairs with the same separation of 0.72 nm, denoted '1' – '4' in Figure S2, using 21 different tips (B_{ext} = 0.9 T, θ_{ext} = 82°). Note that splitting measured from pairs of 0.72 nm scatters in the range of 0.2–1.4 GHz (see also Figure S1 and S2), however, each pair showed a much weaker dependence on the tips used, with a dispersion of ~ 0.1 GHz from its mean splitting. The open circle represents the average splitting of each pair with its error bar.

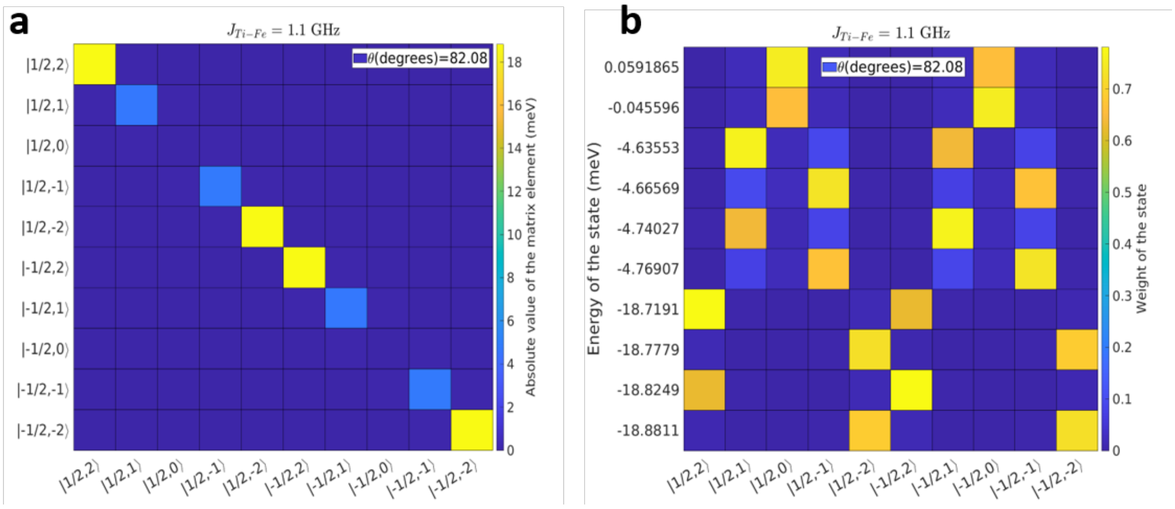

Figure S4. Model Hamiltonian and eigenstates of a Ti-Fe pair. Checkerboard representations of (a) the Hamiltonian Equation S2 for a pair of Ti-Fe separation of 0.72 nm and (b) its eigenstates (B_{ext} = 0.9 T, θ_{ext} = 82°).

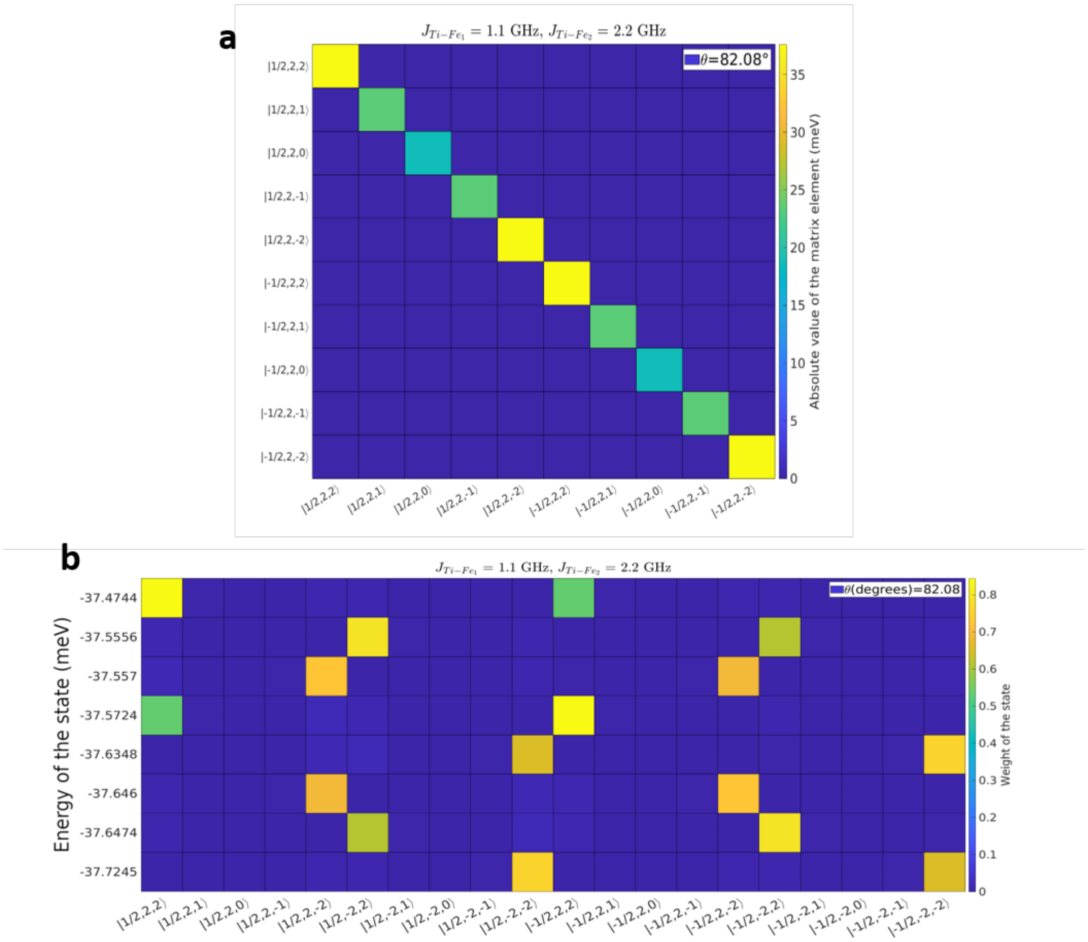

Figure S5. Model Hamiltonian of a Fe-Ti-Fe spin complex. Checkerboard representations of (a) subset of the Hamiltonian matrix for the first Fe (Fe_1) is up (⇑; $m_{\text{Fe1}} = 2$) and (b) the eigenstates of the eight lowest energies. The spin states are labelled according to the array of three spins, $|m_{\text{Ti}}, m_{\text{Fe1}}, m_{\text{Fe2}}\rangle$ (B_{ext} = 0.9 T, θ_{ext} = 82°).

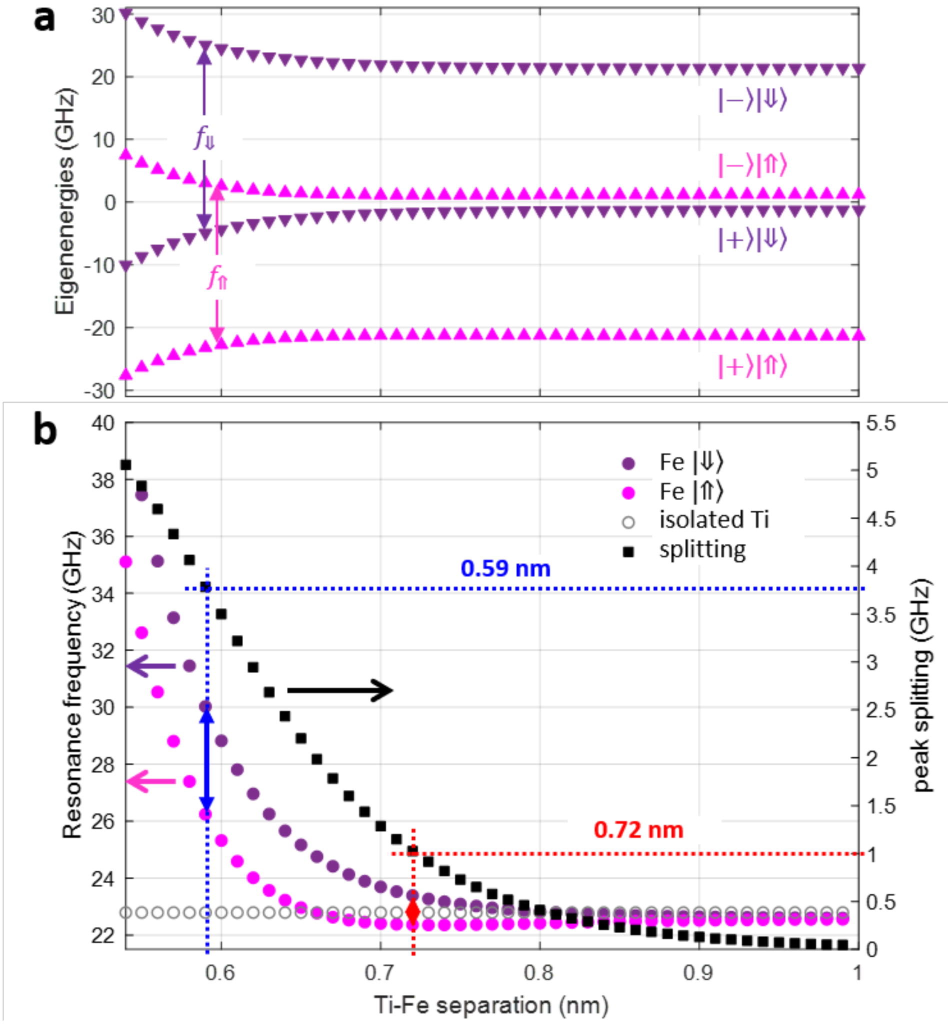

Figure S6. Model simulations of ESR transitions of Ti in a Ti-Fe pair. (a) Eigenstates and (b) peak splitting as a function of Ti-Fe separation using the model Hamiltonian $\widehat{H}_{\mathrm{pair}}$ (Equation S2). The dependence of $J_{\mathrm{Ti,Fe}}$ on the Ti-Fe separation (Figure S1b) was used. In (a), we shifted the four as-calculated eigenenergies by adding the Zeeman energies of the Fe spin for the two ESR transitions ($f_{\Uparrow}$ and $f_{\Downarrow}$) to be clearly visible.

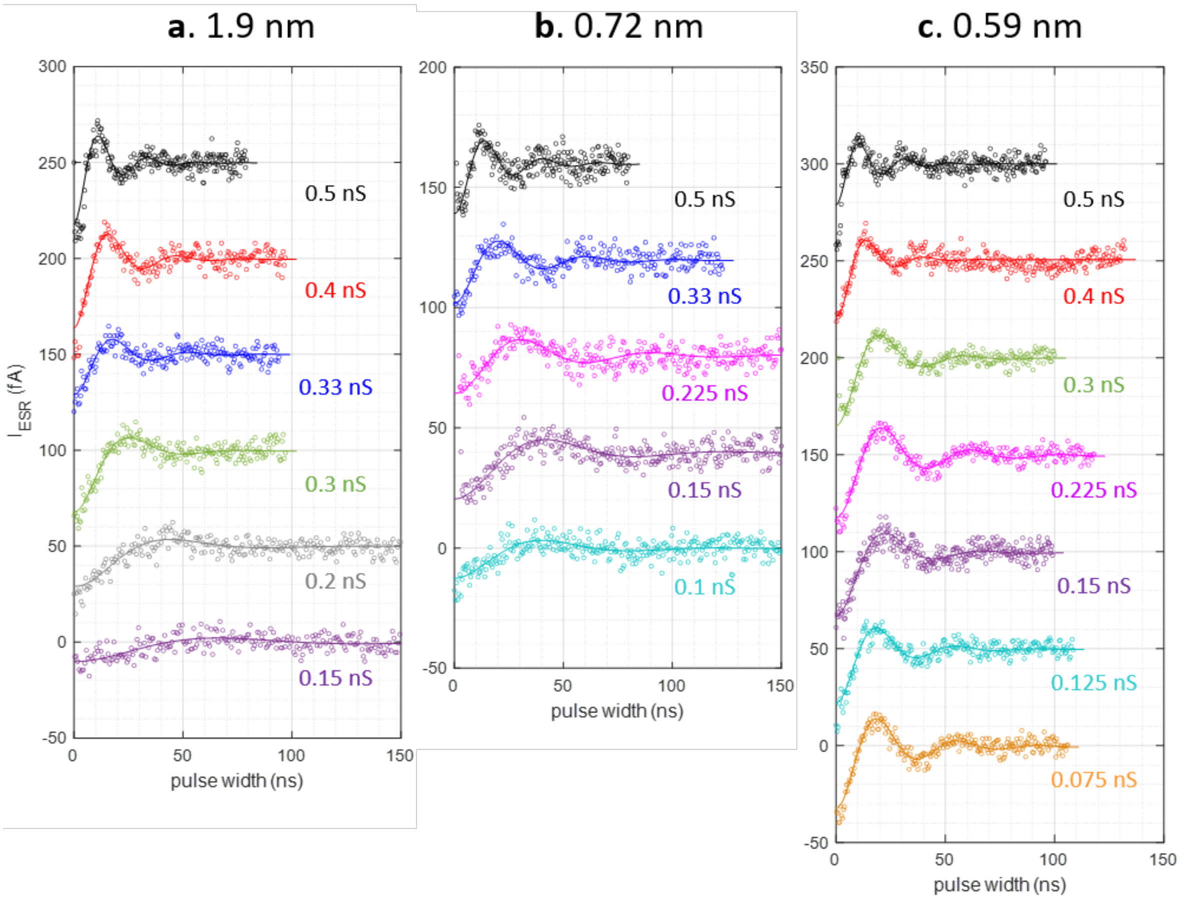

Figure S7. Tunnel conductance dependence of Rabi oscillations measured on the Ti-Fe pairs shown in Figure 1b-d. Solid curves are the fits using an exponentially decaying sinusoidal function, resulting in the Rabi rates in Figure 2c. ($I_{\mathrm{DC}} = 10\ \mathrm{pA}, T = 1.2\ \mathrm{K}, B_{\mathrm{ext}} = 0.9\ \mathrm{T}, \theta_{\mathrm{ext}} = 82°$).

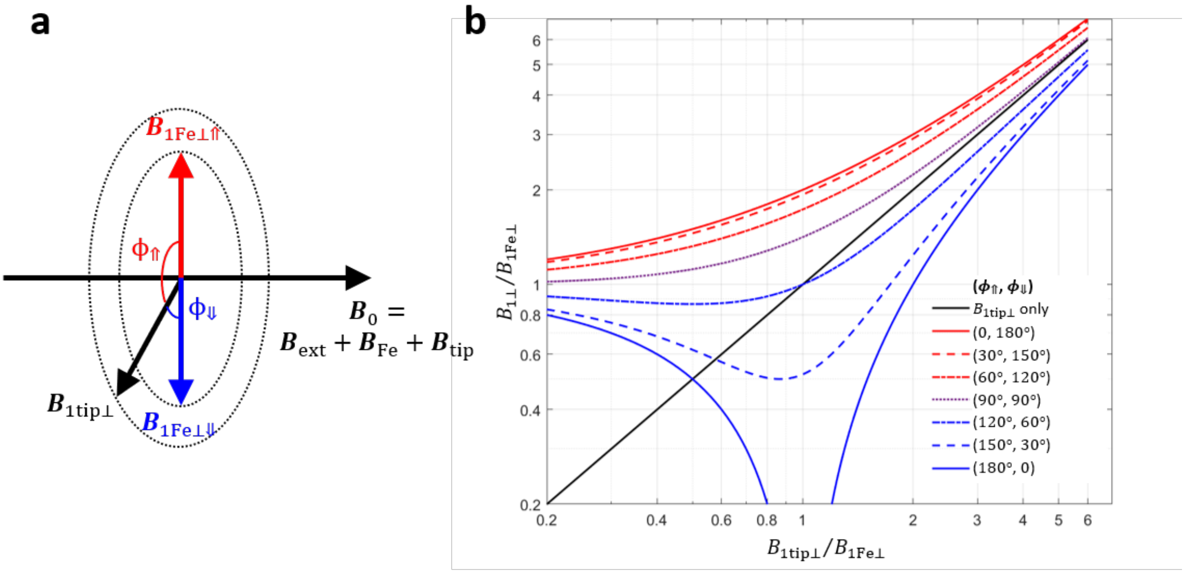

Figure S8. Driving fields in ESR of Ti in a Ti-Fe pair. (a) A schematic illustration of two driving fields ($\boldsymbol{B}_{1,\mathrm{tip}\perp}$, $\boldsymbol{B}_{1,\mathrm{Fe}\perp}$) in ESR of Ti in a Ti-Fe pair. The red and blue arrows denote two Fe-induced driving fields for two quasi-static spin states of Fe, $|\Uparrow\rangle$ and $|\Downarrow\rangle$, respectively. $\boldsymbol{B}_0$ is the total static field applied to the Ti spin, composed of three contributions from external, tip-induced, and Fe-induced fields. (b) Magnitude of total driving field ($B_{1\perp}$) following the Equation S6 as a function of tip-originated driving field ($B_{1,\mathrm{tip}\perp}$), depending on the angle ($\phi_\Uparrow$ or $\phi_\Downarrow$) between $\boldsymbol{B}_{1,\mathrm{tip}\perp}$ and $\boldsymbol{B}_{1,\mathrm{Fe}\perp}$.

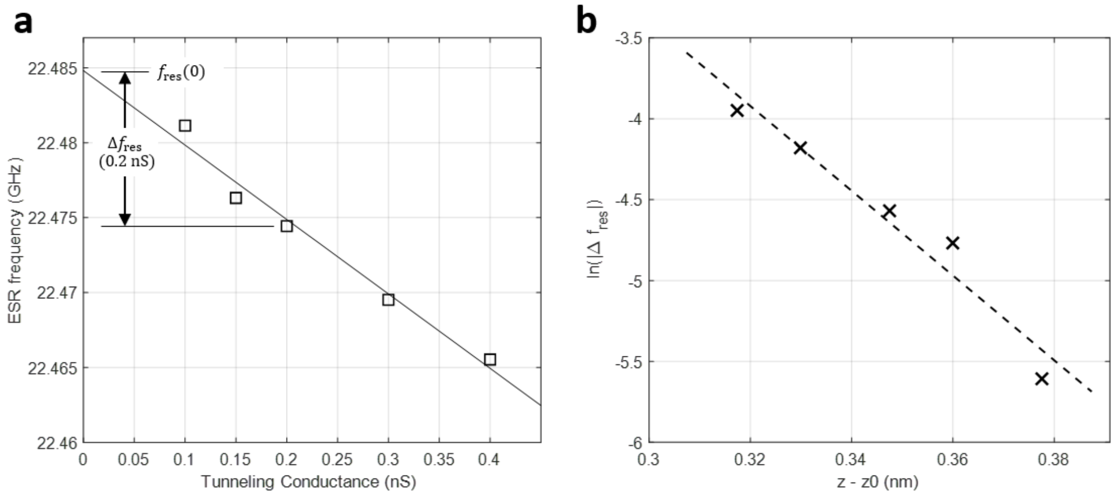

Figure S9. ESR resonance frequency on an isolated Ti. (a) Resonance frequency (f_{res}) as function of tunnel conductance. (b) Shift of f_{res} (Δf_{res}) measured from the f_{res} at zero conductance, as indicated in (a), as a function of tip height (z). Using as-obtained d_0 and σ_0 in the discussion above, we converted the measurement tunnel conductance into the tip height (z). The solid lines are the linear fits of the plots.

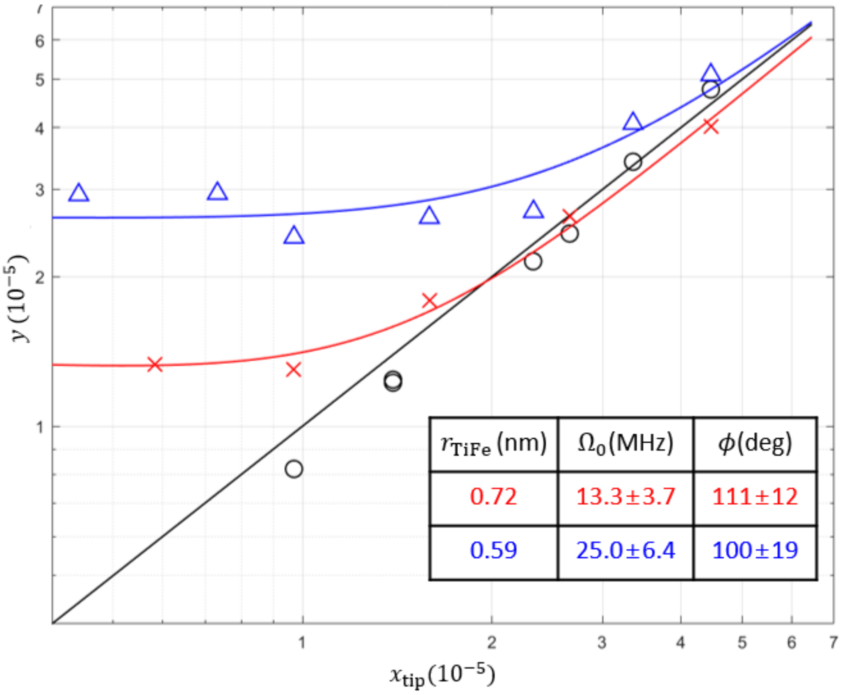

Figure S10. Fits of Rabi rates vs. tunnel conductance for the three cases in Figure 2c. Replot the data in Figure 2c using the model discussed in the above text with the x- and y-axis variables defined in Equation S10 and S11. The fits resulted in the parameters Ω_0 and $\phi_{\Uparrow}$, as shown in the inset.

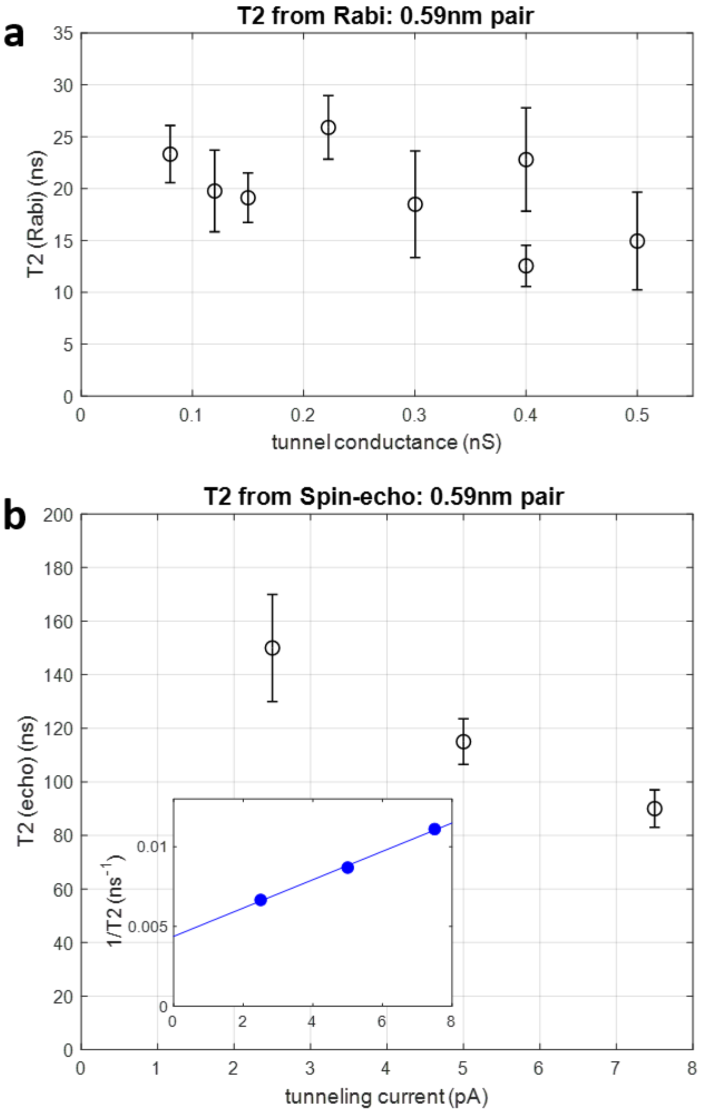

Figure S11. Coherence time of Ti spin measured from the Ti-Fe pair in Figure 2. (a) Coherence times extracted from the Rabi oscillation curves measured on the pair of 0.59 nm (Figure 2c, Figure S7c). (b) Coherence times measured on Ti spin with an Fe atom at a distance of 0.59 nm using the spin-echo measurement scheme. Measurement conditions: $V_{\mathrm{DC}} = 50\ \mathrm{mV}, T = 1.2\ \mathrm{K}, B_{\mathrm{ext}} = 0.9\ \mathrm{T}, \theta_{\mathrm{ext}} = 82°$.

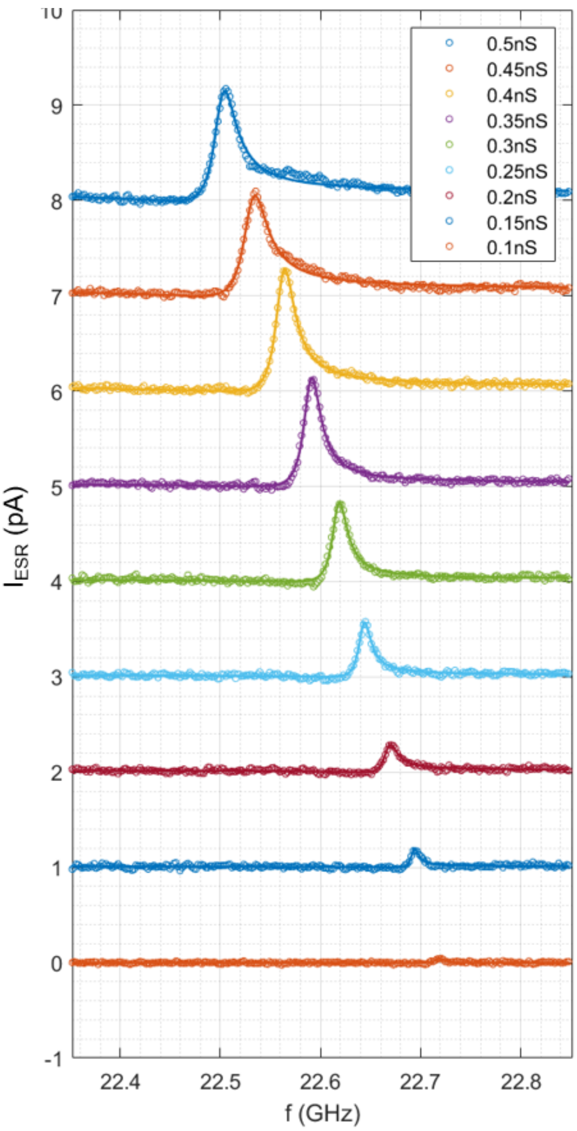

Figure S12. Tunnel conductance dependence of ESR spectra of a single Ti measured with the same tip used in Figure 3. As the tip was retracted from the Ti spin, the peak height monotonically decreases, and it vanishes. ($V_{\mathrm{DC}} = 50\ \mathrm{mV}, T = 1.2\ \mathrm{K}, B_{\mathrm{ext}} = 0.9\ \mathrm{T}, \theta_{\mathrm{ext}} = 82°$).

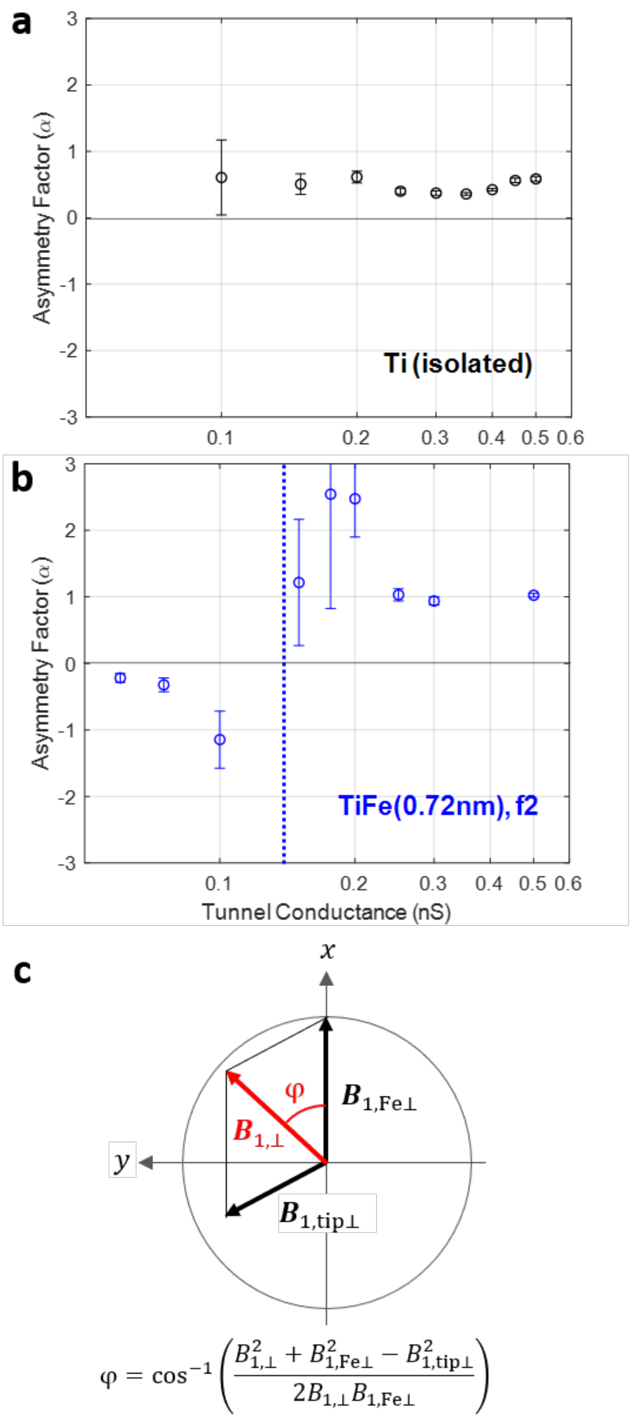

Figure S13. Peak asymmetry of Ti ESR spectra using the same tip in Figure 3. Asymmetry factor (α) extracted from fits of ESR peaks shown in (a) Figure S11 and (b) Figure 3a to a continuous wave STM-ESR peak model (Equation S9 in Reference 23) for a varying tunnel conductance. The vertical dotted line in b indicates the tunnel conductance across which the ESR peak vanishes. (c) Vector diagram of total transverse driving field ($\boldsymbol{B}_{1,\perp}$) composed of the tip ($\boldsymbol{B}_{1,\mathrm{tip},\perp}$) and Fe ($\boldsymbol{B}_{1,\mathrm{Fe},\perp}$) contributions, which are neither parallel nor antiparallel, where the phase shift (φ) is determined by the trigonometric relationship of the three vectors, as denoted.

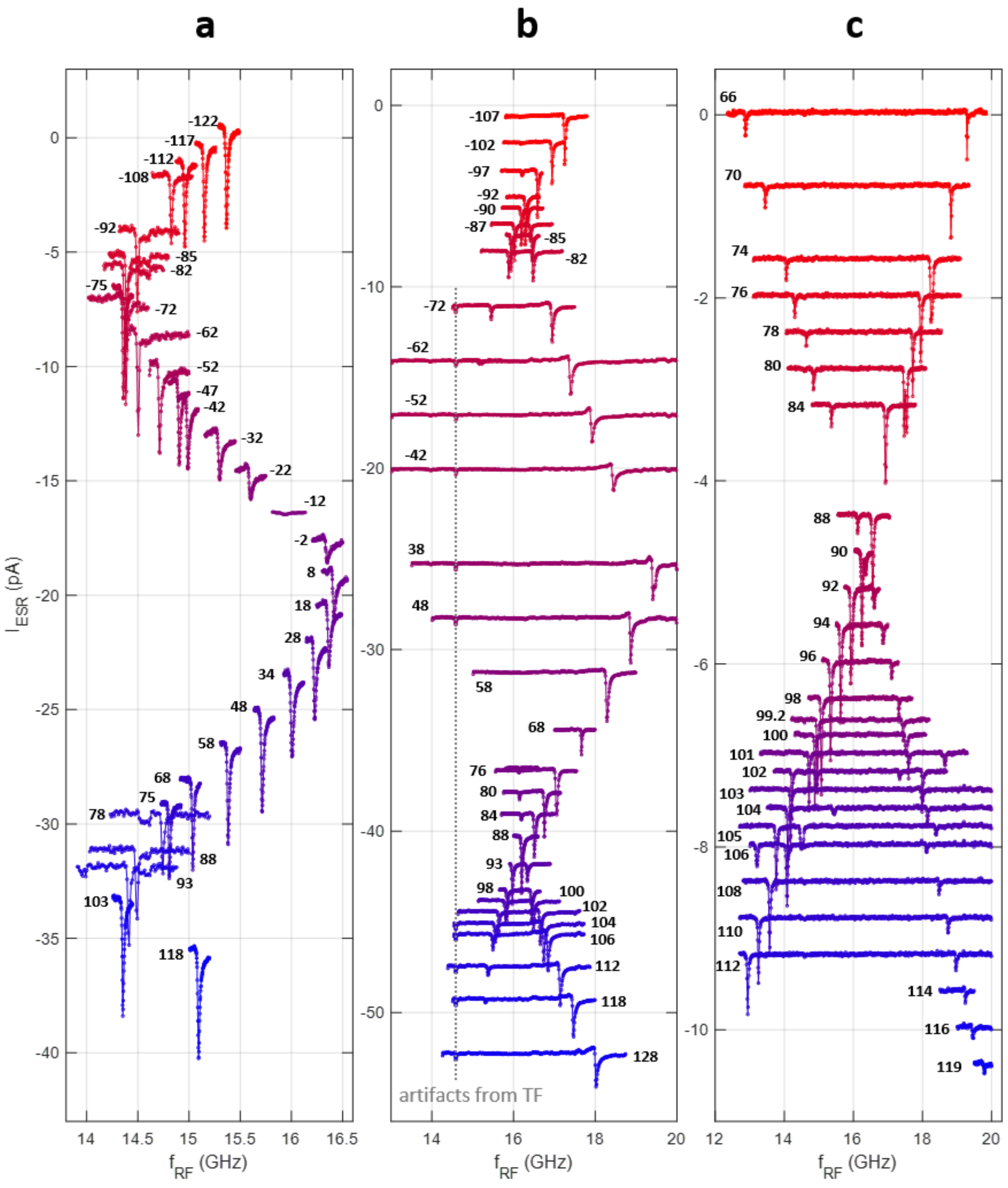

Figure S14. ESR of Ti-Fe pairs with varying angle of external field (θ_{ext}) for the data in Figure 4. ESR spectra on (a) an isolated Ti ($I_{tun} = 10$ pA, $V_{DC} = 30$ mV, $V_{RF} = 30$ mV) and (b and c) Ti-Fe pairs with separations of 0.72 nm ($I_{tun} = 20$ pA, $V_{DC} = 30$ mV, $V_{RF} = 30$ mV) and 0.59 nm ($I_{tun} = 10$ pA, $V_{DC} = 200$ mV, $V_{RF} = 30$ mV), respectively. Each spectrum is indicated by the polar angle, in degree (°), of the external field used in measurement and presented with a I_{ESR}-offset for clarity. Gray dotted line indicates artifacts from the transfer function (TF) of the RF transmission at 14.6 GHz.

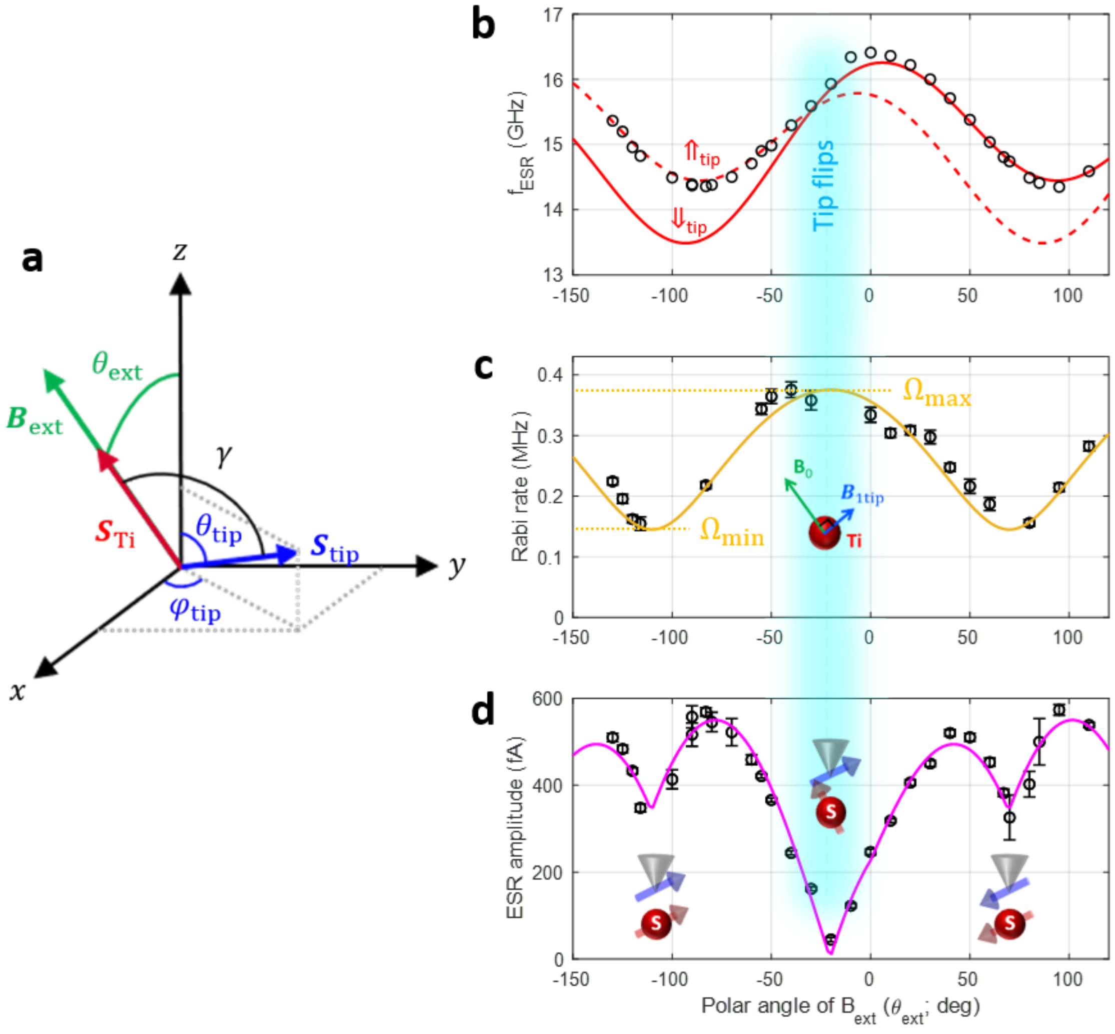

Figure S15. Analysis of data from an isolated Ti spin. (a) A schematic showing the definitions of the angles used in the model. Plots of (b) ESR frequencies, (c) Rabi rates, and (d) ESR amplitudes measured from an isolated Ti spin as a function of the polar angle of the external field (θ_{ext}) ($I_{tun} = 10$ pA, $V_{DC} = 30$ mV). Solid curves are the fits using the model discussed in the text.

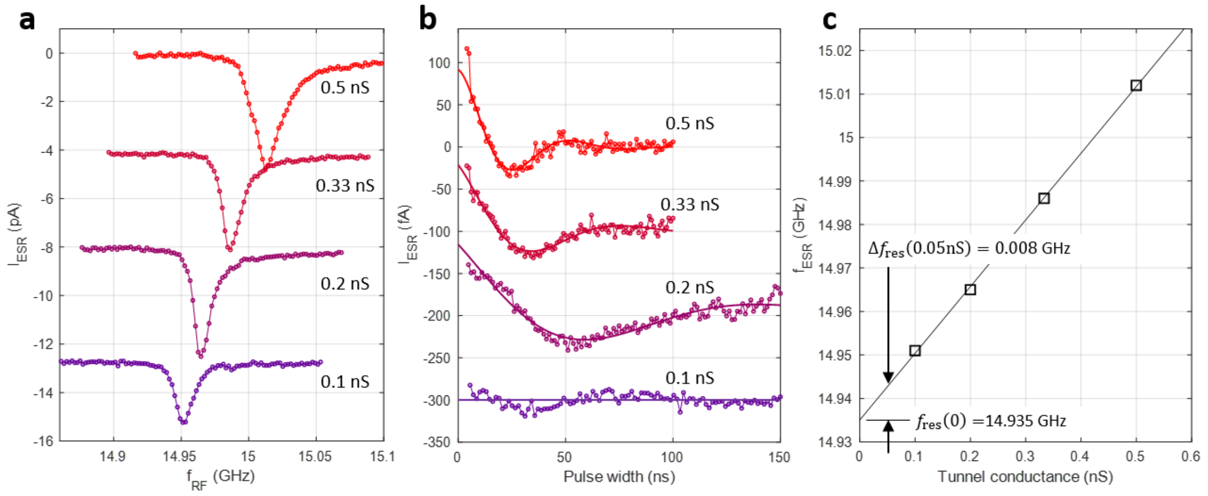

Figure S16. Tunnel conductance dependence of CW- and pulsed-ESR on an isolated Ti. (a) CW-ESR spectra and (b) Rabi oscillations measured on the same tip and Ti atom as used for the data in Figure S13. (c) ESR resonance frequencies extracted from the spectra in (a). Solid line is a linear fit. ($I_{\mathrm{DC}} = 10$ pA, $T = 0.4$ K, $B_{\mathrm{ext}} = 0.6$ T, $\theta_{\mathrm{ext}} = 72°$).

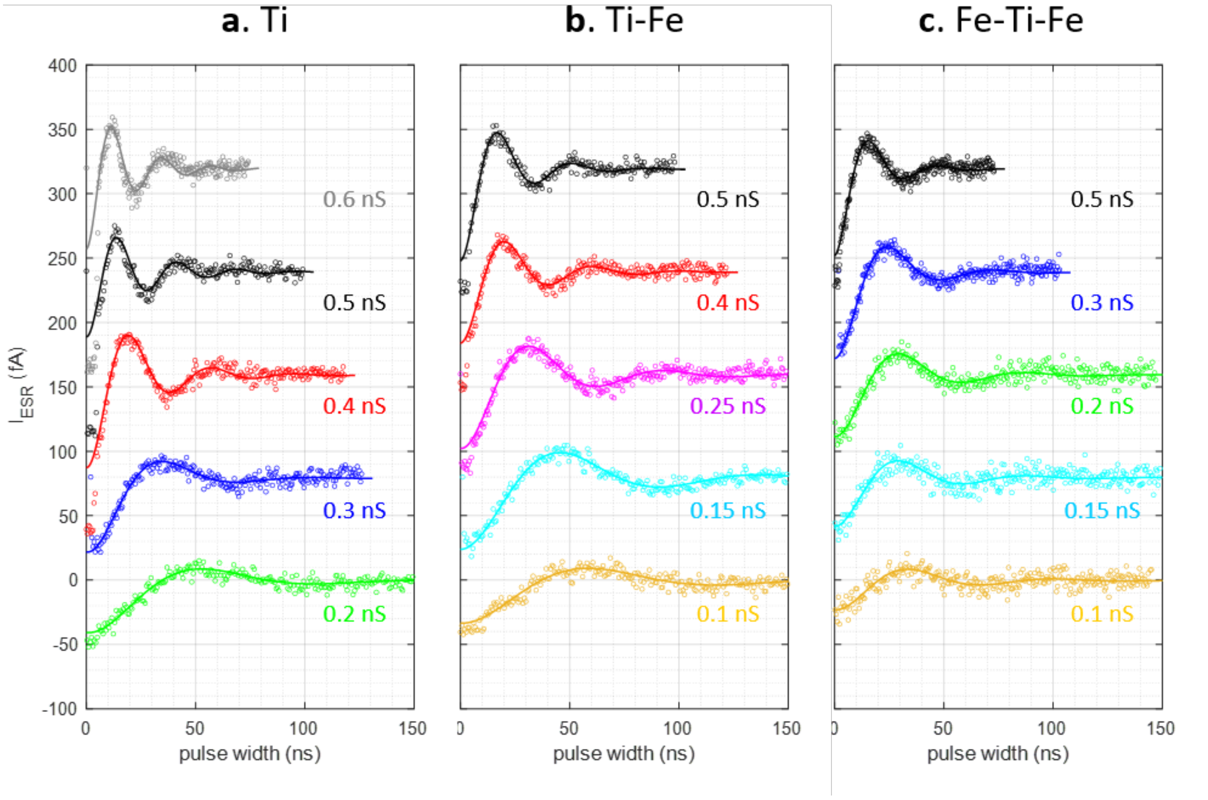

Figure S17. Tunnel conductance dependence of Rabi oscillations measured on the Fe-Ti-Fe complex shown in Figure 5. Solid curve are the fits using an exponentially decaying sinusoidal function, giving the Rabi rates Ω shown in Figure 5c. ($I_{\mathrm{DC}} = 10$ pA, $T = 0.6$ K, $B_{\mathrm{ext}} = 0.9$ T, $\theta_{\mathrm{ext}} = 82°$).

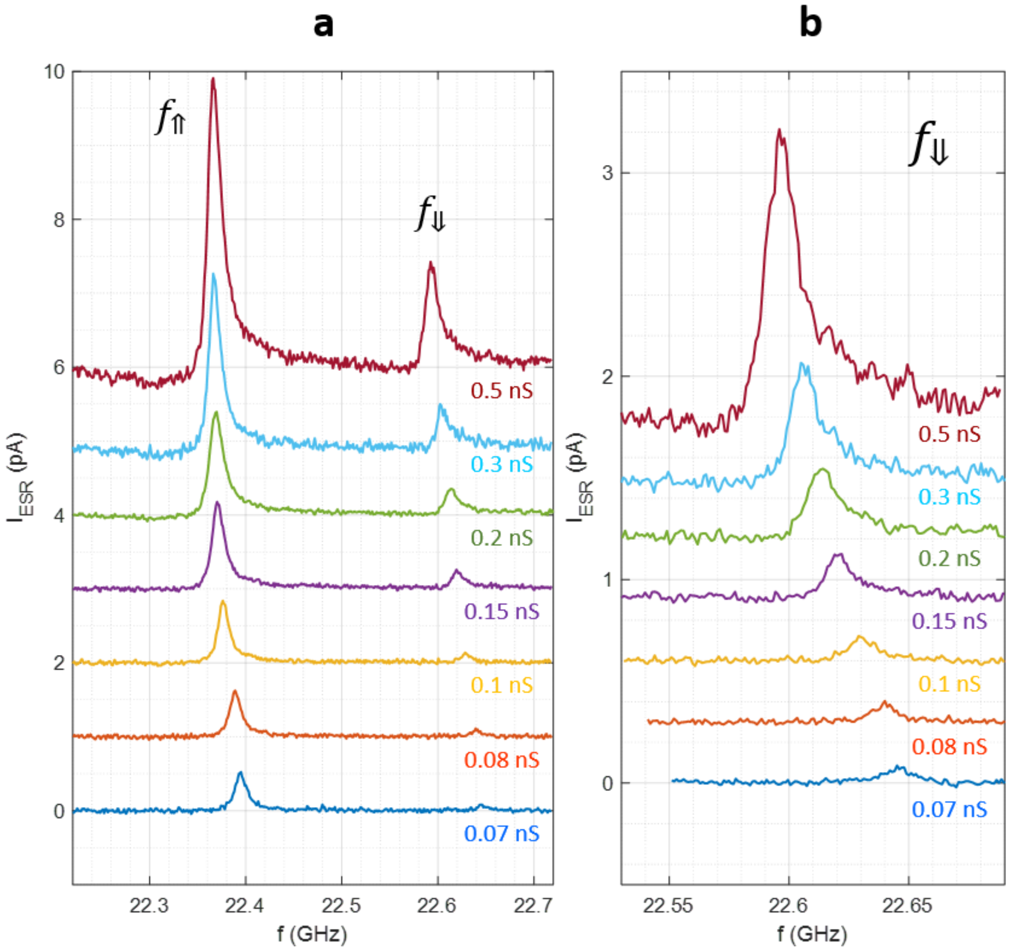

Figure S18. Tunnel conductance dependence of ESR spectra on a Ti-Fe pair. (a) ESR spectra showing both lower ($f_\Uparrow$) and higher ($f_\Downarrow$) resonances. (b) Zoom-in across the peak at the higher resonance ($f_\Downarrow$). Note that both peaks showed monotonic decrease of the height as the tip was moved out from the Ti spin, in a tunnel conductance regime comparable to that in Figure 3 ($I_{\mathrm{DC}} = 10$ pA, $T = 1.2$ K, $B_{\mathrm{ext}} = 0.9$ T, $\theta_{\mathrm{ext}} = 82°$).

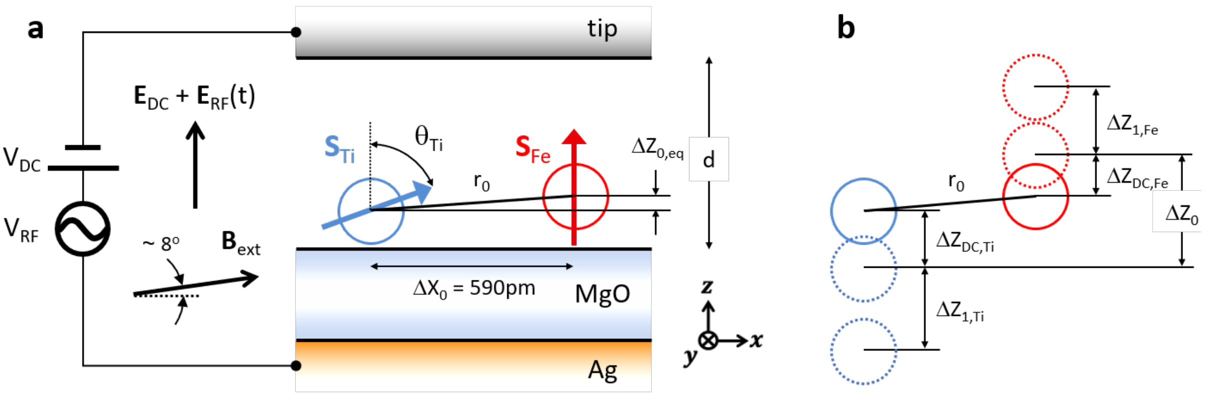

Figure S19. Schematic of a Ti-Fe pair in the tunnel junction to derive piezoelectric motions of the spins. (a) The geometry of the tip-substrate tunnel junction is approximated to a parallel plate capacitor, where the DC and RF biases are applied to the substrate (Ag) in series while the tip is grounded. (b) An illustration of piezoelectric displacements of the atoms in opposite phases (*6*) under the DC and RF electric fields shown in (a). The vertical scale for the displacements of the atoms are exaggerated compared to the horizontal scale for clarity.

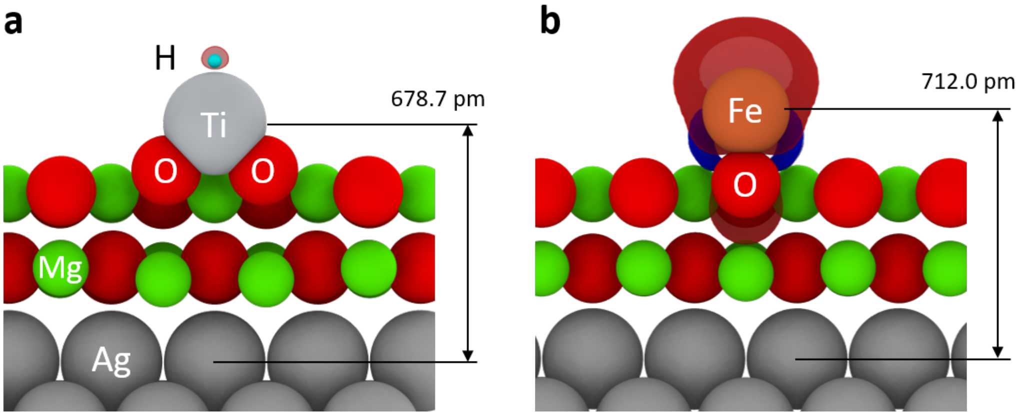

Figure S20. DFT-calculated geometries of Ti (a) and Fe (b) atoms adsorbed on 2ML-thick MgO on Ag(100).
